# SMART: An Open-Source Extension of WholeBrain for Intact Mouse Brain Registration and Segmentation

**DOI:** 10.1523/ENEURO.0482-21.2022

**Published:** 2022-05-03

**Authors:** Michelle Jin, Joseph D. Nguyen, Sophia J. Weber, Carlos A. Mejias-Aponte, Rajtarun Madangopal, Sam A. Golden

**Affiliations:** 1 Intramural Research Program, National Institute on Drug Abuse, National Institutes of Health, Baltimore 21224, MD; 2 Department of Biological Structure, University of Washington, Seattle 98195, WA

**Keywords:** activity mapping, brain-wide activity mapping, immediate early gene, light sheet microscopy, neuronal ensembles, tissue clearing

## Abstract

Mapping immediate early gene (IEG) expression across intact mouse brains allows for unbiased identification of brain-wide activity patterns underlying complex behaviors. Accurate registration of sample brains to a common anatomic reference is critical for precise assignment of IEG-positive (“active”) neurons to known brain regions of interest (ROIs). While existing automated voxel-based registration methods provide a high-throughput solution, they require substantial computing power, can be difficult to implement and fail when brains are damaged or only partially imaged. Additionally, it is challenging to cross-validate these approaches or compare them to any preexisting literature based on serial coronal sectioning. Here, we present the open-source R package SMART (Semi-Manual Alignment to Reference Templates) that extends the WholeBrain R package framework to automated segmentation and semi-automated registration of intact mouse brain light-sheet fluorescence microscopy (LSFM) datasets. The SMART package was created for novice programmers and introduces a streamlined pipeline for aligning, registering, and segmenting LSFM volumetric datasets across the anterior-posterior (AP) axis, using a simple “choice game” and interactive menus. SMART provides the flexibility to register whole brains, partial brains or discrete user-chosen images, and is fully compatible with traditional sectioned coronal slice-based analyses. We demonstrate SMART’s core functions using example datasets and provide step-by-step video tutorials for installation and implementation of the package. We also present a modified iDISCO+ tissue clearing procedure for uniform immunohistochemical labeling of the activity marker Fos across intact mouse brains. The SMART pipeline, in conjunction with the modified iDISCO+ Fos procedure, is ideally suited for examination and orthogonal cross-validation of brain-wide neuronal activation datasets.

## Significance Statement

Immediate early genes (IEG) are invaluable for revealing the structure-activity-function relationships of different brain regions. By leveraging the activity-dependent expression of IEGs (e.g., Fos), brain activity in mice can be associated with recent experiences. The combination of advances in tissue clearing, immunolabelling, and volumetric fluorescent imaging provide the methodology to extract brain-wide imaging datasets of IEG expression. Automated pipelines can analyze these datasets, but often exclude accessible validation checkpoints. There is a need for computational tools that streamline analysis of such datasets and include human checkpoints for orthogonal cross-validation of automated approaches. Addressing this gap, we introduce SMART, an open-source user-friendly R package, which builds on existing registration and automated cell counting methods, to facilitate whole-brain IEG mapping in mice.

## Introduction

Neural activity mapping using immediate early gene (IEG) expression is invaluable for the identification of brain-wide activity patterns underlying behavior (Renier et al., 2016; Ye et al., 2016). Recent advances in whole-mount tissue clearing and immunohistochemistry (IHC; Ertürk et al., 2011, 2012; Renier et al., 2014; Susaki et al., 2014; Lee et al., 2016; Ueda et al., 2020b), coupled with improvements in volumetric fluorescent imaging (Reynaud et al., 2008; Power and Huisken, 2017) and open-source brain mapping pipelines (Renier et al., 2016; Fürth et al., 2018), have made it possible to analyze IEG expression across intact brains and identify novel structure-activity-function relationships at multiple spatial scales (DeNardo et al., 2019; Franceschini et al., 2020; Kimbrough et al., 2020; Ueda et al., 2020a).

A key technical challenge is the precise registration of experimental brains to a common reference atlas before assignment of detected IEG-positive (“active”) neurons to known anatomic regions of interest (ROIs). Existing automated voxel-based registration methods provide a high-throughput solution, but require substantial computing power, are challenging for novice programmers, and fail when brains are incomplete or damaged during staining. Additionally, these approaches provide regional counts spanning the entire anterior-posterior (AP) axis and are challenging to cross-validate or compare against classical “coronal-chunk” activity mapping studies using serial sections around ROIs (DeNardo et al., 2019; Kimbrough et al., 2020, 2021).

The R package WholeBrain (Fürth et al., 2018) provides an elegant alternative that combines semi-automated user-refinable registration with automated segmentation of vectorized coronal reference atlas plates and can accommodate flexible imaging resolutions. However, WholeBrain is not optimized for registration of entire mouse brain imaging datasets for three main reasons. First, users are required to manually identify anatomic coordinates along the AP axis that correspond to the reference atlas plate. Second, as segmentation is performed on individual 2D images and then assigned to 3D space, for datasets with a high-resolution z-step size, segmentation can result in duplicate counts across adjacent images. Third, WholeBrain does not account for nonuniformities in tissue morphing along the AP axis during staining and clearing when making z assignments.

To address these gaps, we developed an open-source R package called SMART (Semi-Manual Alignment to Reference Templates), as an extension to the existing WholeBrain package. SMART provides a streamlined pipeline for registration, segmentation and analysis of high-resolution partial and whole-brain imaging datasets. SMART is built with the novice programmer in mind, includes a console interface to guide users through analysis and visualization, and requires minimal programming experience. Notably, SMART allows registration of discrete, user-chosen image stacks across a mouse brain (termed partial datasets) and provides regional counts that can be directly compared against studies based on coronally sectioned brain slice imaging datasets (Crombag et al., 2002; Koya et al., 2009; Bossert et al., 2011, 2012; Navailles et al., 2015; Pelloux et al., 2018; Campbell et al., 2019; Warren et al., 2019; Kane et al., 2021).

Here, we demonstrate the key features of SMART using example mouse whole-brain activity-mapping datasets. We present a modified version of the iDISCO+ immunolabeling and clearing protocol for uniform whole-brain Fos labeling, illustrate the application of SMART to user-guided registration and segmentation of these datasets, and demonstrate new visualization tools for graphical representation of detected Fos-positive (behaviorally active) cells. The goal of SMART is to make brain-wide activity analysis accessible to researchers with limited computational infrastructure and programming expertise. To this end, we provide a static docker image of SMART so that new users can test out SMART’s core functions before installing WholeBrain and SMART. We also provide a dedicated webpage with detailed installation instructions, step-by-step video tutorials, and a sample dataset (both raw and fully analyzed data) as a training resource for new users. We discuss the utility of SMART for expert cross-validation of automated whole-brain analysis pipelines such as ClearMap.

## Materials and Methods

### Subjects and behavior

We used two three- to six-month-old CD-1 male mice (Charles River Labs, CD-1 IGS; strain code 022) and one Thy-1 GFP transgenic (The Jackson Laboratory, B6; CBA-Tg (Thy1-EGFP) SJrs/NdivJ; stock 011070) mouse that we maintained on a reverse 12/12 h light/dark cycle (lights off at 8 A.M.). We performed all surgical procedures in accordance with the *Guide for the Care and Use of Laboratory Animals* (Ed 8, 2011), under protocols approved by the local Animal Care and Use Committee. CD-1 mice used for example datasets were taken from a cohort that underwent operant aggression self-administration as previously described (Golden et al., 2017, 2019a,b), Mouse 1 (control) was perfused directly from home cage and Mouse 2 (test) was perfused 90 min after a reinforced aggression test.

### Tissue clearing and immunolabeling using modified iDISCO+

We used a modified version of the iDISCO+ protocol to achieve uniform Fos immunostaining and tissue clearing across intact mouse brains. Details of the sample pretreatment, immunolabeling and clearing steps are provided in the sections below. The timeline for the entire procedure is shown in Table 1, and a list of reagents and suggested suppliers is shown in Table 2. We also indicate time ranges where appropriate to allow flexibility in scheduling.

**Table 1:** Table of step-by-step instructions for our modified version of the iDISCO+ immunolabelling and tissue clearing protocol for staining Fos in intact mouse brains

Step #	Chemical	Time	Temperature	Container	Notes
1a	First PBS, then 4% PFA	-	-	-	Transcardial perfusion
1b	4% PFA	Overnight	4°C	15-ml Falcon tube	
1c	PBS	30–60 min × 3 times	RT	15-ml Falcon tube	Mix using a rotary shaker
Note: Prior to the next step, samples can be transferred to PBS with 0.1% sodium azide and stored long term at 4°C if necessary					

2a	100% dH_2_O	1 h × 2	RT	15-ml Falcon tube	Mix using a rotary shaker
2b	20% MeOH in dH_2_O	1.5 h	RT	15-ml Falcon tube	Mix using a rotary shaker
2c	40% MeOH in dH_2_O	1.5 h	RT	15-ml Falcon tube	Mix using a rotary shaker
2d	60% MeOH in dH_2_O	1.5 h	RT	15-ml Falcon tube	Mix using a rotary shaker
2e	80% MeOH in dH_2_O	1.5 h	RT	15 ml Falcon tube	Mix using a rotary shaker
2f	100% MeOH	1.5 h	RT	15-ml Falcon tube	Mix using a rotary shaker
2g	100% MeOH	Overnight	RT	15-ml Falcon tube	Mix using a rotary shaker
3a	66% DCM + 33% MeOH	2 h	RT	15-ml Falcon tube	Mix using a rotary shaker and wait till the brain sinks to the bottom
3b	66% DCM + 33% MeOH	Overnight	RT	15-ml Falcon tube	Mix using a rotary shaker and wait till the brain sinks to the bottom
3c	100% MeOH	3 h	RT	15-ml Falcon tube	Mix using a rotary shaker
4a	100% MeOH	3 h	RT	15-ml Falcon tube	Mix using a rotary shaker
4b	5% H_2_O_2_ in 100% MeOH	Overnight	4°C	15-ml Falcon tube	Mix using a rotary shaker
4c	80% MeOH in dH_2_O	1.5 h	RT	15-ml Falcon tube	Mix using a rotary shaker
4d	60% MeOH in dH_2_O	1.5 h	RT	15-ml Falcon tube	Mix using a rotary shaker
4e	40% MeOH in dH_2_O	1.5 h	RT	15-ml Falcon tube	Mix using a rotary shaker
4f	20% MeOH in dH_2_O	1.5 h	RT	15-ml Falcon tube	Mix using a rotary shaker
4g	100% dH_2_O	1.5 h	RT	15-ml Falcon tube	Mix using a rotary shaker
5a	PBS	1 h	RT	15-ml Falcon tube	Mix using a rotary shaker
5b	PTx0.5 (PBS with 0.5% Triton X-100)	1 h	37°C	15-ml Falcon tube	Mix using a rotary shaker
5c	PTx0.5	1 h	37°C	15-ml Falcon tube	Mix using a rotary shaker
5d	PTx0.5	Overnight	37°C	15-ml Falcon tube	Mix using a rotary shaker
Note: Prior to the next step, samples can be transferred to PTx0.5 with 0.1% sodium azide and stored for a few days at 37°C if necessary					

6a	Permeabilization buffer (78.6% PTx0.5 + 1.4% glycine + 20% DMSO)	48 h	37°C	15-ml Falcon tube	Mix using a rotary shaker; incubation time can be extended by 24–48 h if needed
6b	Blocking buffer (84% PTx0.5 + 6% NDS + 10% DMSO)	48 h	37°C	15-ml Falcon tube	Mix using a rotary shaker; incubation time can be extended by 24–48 h if needed
Note: For the next step, we observed that five sequential additions of 1° antibody at 1:1000 dilution (1° Ab booster) helped improve penetration and labeling over a single bolus addition at a 1:200 dilution					

7a	1° Antibody (Ab) buffer (92% PTwH0.5 + 3% NDS + 5% DMSO) with 1:1000 1° Ab	24 h	37°C	1.2-ml Corning cryovial with external threads	Mix using a rotary shaker
7b	1° Ab booster (1:1000 addition of 1° Ab)	24 h	37°C	1.2-ml Corning cryovial with external threads	Mix using a rotary shaker
7c	1° Ab booster	24 h	37°C	1.2-ml Corning cryovial with external threads	Mix using a rotary shaker
7d	1° Ab booster	24 h	37°C	1.2-ml Corning cryovial with external threads	Mix using a rotary shaker
7e	1° Ab booster	72 h	37°C	1.2-ml Corning cryovial with external threads	Mix using a rotary shaker
8a	PTwH0.5 (PBS with 0.5% Tween 20 + 10 μg/ml heparin)	24 h	37°C	15-ml Falcon tube	Mix using a rotary shaker
8b	PTwH0.5	24 h	37°C	15-ml Falcon tube	Mix using a rotary shaker
8c	PTwH0.5	24 h	37°C	15-ml Falcon tube	Mix using a rotary shaker
8d	PTwH0.5	24 h	37°C	15-ml Falcon tube	Mix using a rotary shaker
Note: Prior to the next step, samples can be transferred to PTwH0.5 with 0.1% sodium azide and stored for a few days at 37°C if necessary; for the next step, we observed that five sequential additions of 2° antibody at 1:500 dilution (2° Ab booster) helped improve penetration and labeling over a single bolus addition at a 1:100 dilution					

9a	2° Ab buffer (97% PTwH0.5 + 3% NDS) with 1:500 2° Ab	24 h	37°C	1.2-ml Corning cryovial with external threads	Mix using a rotary shaker
9b	2° Ab booster (1:500 addition of 2° Ab)	24 h	37°C	1.2-ml Corning cryovial with external threads	Mix using a rotary shaker
9c	2° Ab booster	24 h	37°C	1.2-ml Corning cryovial with external threads	Mix using a rotary shaker
9d	2° Ab booster	24 h	37°C	1.2-ml Corning cryovial with external threads	Mix using a rotary shaker
9e	2° Ab booster	48 h	37°C	1.2-ml Corning cryovial with external threads	Mix using a rotary shaker
10a	PTwH0.5	24 h	37°C	15-ml Falcon tube	Mix using a rotary shaker
10b	PTwH0.5	24 h	37°C	15-ml Falcon tube	Mix using a rotary shaker
10c	PTwH0.5	24 h	37°C	15-ml Falcon tube	Mix using a rotary shaker
10d	PTwH0.5	24 h	37°C	15-ml Falcon tube	Mix using a rotary shaker
Note: Prior to the next step, samples can be transferred to PBS with 0.1% sodium azide and stored long term at 4°C if necessary					

11a	100% dH_2_O	1 h	RT	15-ml Falcon tube	Mix using a rotary shaker
11b	20% MeOH in dH_2_O	1.5 h	RT	15-ml Falcon tube	Mix using a rotary shaker
11c	40% MeOH in dH_2_O	1.5 h	RT	15-ml Falcon tube	Mix using a rotary shaker
11d	60% MeOH in dH_2_O	1.5 h	RT	15-ml Falcon tube	Mix using a rotary shaker
11e	80% MeOH in dH_2_O	1.5 h	RT	15-ml Falcon tube	Mix using a rotary shaker
11f	100% MeOH	1.5 h	RT	15-ml Falcon tube	Mix using a rotary shaker
12a	100% MeOH	1.5 h	RT	15-ml Falcon tube	Mix using a rotary shaker
12b	66% DCM + 33% MeOH	2 h	RT	15-ml Falcon tube	Mix using a rotary shaker and wait till the brain sinks to the bottom
12c	66% DCM + 33% MeOH	2 h	RT	15-ml Falcon tube	Mix using a rotary shaker and wait till the brain sinks to the bottom
12d	66% DCM + 33% MeOH	Overnight	RT	15-ml Falcon tube	Mix using a rotary shaker and wait till the brain sinks to the bottom
12e	100% DCM	3 h	RT	Wide mouth amber vial with PTFE lined cap	Mix using a rotary shaker and wait till the brain sinks to the bottom
12f	100% DCM	3 h	RT	Wide mouth amber vial with PTFE lined cap	Mix using a rotary shaker and wait till the brain sinks to the bottom
13a	DBE	3 h	RT	Wide mouth amber vial with PTFE lined cap	Mix using a rotary shaker and wait till the brain sinks to the bottom
13b	DBE	3 h	RT	Wide mouth amber vial with PTFE lined cap	Mix using a rotary shaker and wait till the brain sinks to the bottom
13c	DBE	3 h	RT	Wide mouth amber vial with PTFE lined cap	Keep stationary
14	Intact brain imaging using LSFM				

Times recommended for each step are listed along with sample preparation details and conditions.

**Table 2:** List of the reagents and antibodies used in our modified version of the iDISCO+ protocol for staining Fos in intact mouse brains

Reagent	Details	Supplier
Phospho-c-Fos (Ser32) (D82C12) XP rabbit mAb	5348S (lot #1)	Cell Signaling
Anti-GFP/YFP (GFP antibody)	GFP-1020 (lot #GFP697986)	Aves Laboratory
Alexa Fluor 647 AffiniPure F(ab')^2^, fragment donkey anti-rabbit IgG (H+L)	711-606-152 (lot #128806)	Jackson ImmunoResearch
Alexa Fluor 488 AffiniPure F(ab')^2^, fragment donkey anti-chicken IgY (IgG; H+L)	703-546-155 (lot #127495)	Jackson ImmunoResearch
10× PBS	119-069101	Quality Biological
PFA (prilled)	19202	Electron Microscopy Science
Sodium phosphate (monobasic monohydrate)	BDH9298	VWR Life Science
Tween 20	P9416	Sigma-Aldrich
Triton X-100	X100	Sigma-Aldrich
DCM	270997	Sigma-Aldrich
MeOH	A412SK	Fisher
DMSO	D128-4	Fisher Scientific
Glycine	G7126	Sigma-Aldrich
Heparin	H3393	Sigma-Aldrich
Sodium azide	58032	Sigma-Aldrich
H_2_O_2_ 30%	216763	Sigma
NDS	017-000-121	Jackson ImmunoResearch
Benzyl ether 98%	10801	Sigma-Aldrich
Falcon 15-ml centrifuge tubes (polystyrene)	05-527-90	Fisher Scientific
Wide mouth amber vial (26 × 26 mm)	CT242626-A-TEF-144	Discount Vials
Corning 1.2-ml cryogenic vials with external thread	CLS430658-500EA	Sigma-Aldrich

Supplier and catalog number are listed; lot numbers are provided when possible.

### Sample collection

We anesthetized the mice with isoflurane and perfused transcardially with 200 ml of 0.1 m PBS (pH 7.4) followed by 400 ml of 4% paraformaldehyde (PFA) in PBS (4% PFA, pH 7.4). We extracted brains and postfixed them in 4% PFA (4°C, overnight). We then transferred brains to 15 ml conical polystyrene centrifuge tubes containing PBS with 0.1% sodium azide for long-term storage at 4°C or processed them on the next day.

### Sample pretreatment with methanol (MeOH)

All sample pretreatment steps were performed in 15 ml conical tubes while gently mixing on a rotating mixer (Daigger Scientific, EF24935) and sample tubes were filled to the top with solutions to prevent oxidation. Following equilibration to room temperature (RT), we first washed brains in PBS (RT, 3 × 30 min). We then dehydrated samples using ascending concentrations of MeOH in deionized H_2_O (dH_2_O), 0%, 20%, 40%, 60%, 80%, 100%, 100% MeOH (RT, 1.5 h each). Next, we performed delipidation using 66% dichloromethane (DCM)/33% MeOH (RT, 1 × 8 h followed by overnight). We then washed samples in 100% MeOH (RT, 2 × 3 h each), before bleaching in a chilled hydrogen peroxide (H_2_O_2_)_/_H_2_O/MeOH solution (1 volume 30% H_2_O_2_ to 5 volumes 100% MeOH) overnight at 4°C. Next, we rehydrated samples using descending concentrations of MeOH in dH_2_O, 80%, 60%, 40%, 20%, 0% MeOH (RT, 1.5 h each). We then washed the samples first in PBS at RT (1 × 1 h), then twice in a buffer containing PBS with 0.5% Triton X-100 (PTx0.5) at 37°C (2 × 1 h, followed by 1 × overnight).

### Immunolabeling

Immunolabeling was performed in 1.5-ml Nalgene cryotubes while gently mixing on the rotating mixer and samples were transferred to 15-ml conical centrifuge tubes for permeabilization, blocking and wash steps. We used a buffer containing PBS with 0.5% Tween 20, and 10 μg/ml heparin (PTwH0.5) for all wash steps and sample containers were filled to the top with solutions to prevent oxidation. We first incubated samples in permeabilization buffer containing 78.6% PTx0.5, 1.4% glycine, and 20% dimethyl sulfoxide (DMSO), and then in blocking buffer containing 84% PTx0.5, 6% normal donkey serum (NDS), and 10% DMSO (37°C, 2 d each). Next, we incubated samples in primary (1°) antibodies [anti-cFos: phospho-c-Fos (Ser32) (D82C12) XP Rabbit mAb, Cell Signaling Technology, #5348S Lot 1; anti-GFP, RRID:AB_10557109; green fluorescent protein (GFP) Antibody, Aves Labs GFP-1020, lot #GFP697986, RRID:AB_10000240] diluted in 1° antibody buffer containing 92% PTwH0.5, 3% NDS, and 5% DMSO (37°C, 7 d). We started with a 1:1000 dilution of antibody on day 1 and supplemented with four equal booster doses of 1° antibody (4 × 1 d) for a final dilution of 1:200 on day 5. After one week of 1° antibody treatment (five additions over 5 d plus two extra days), we performed washes in PTwH0.5 over 4 d (37°C, 4 × 12 h followed by 2 × 1 d). We then incubated samples in secondary (2°) antibodies [Alexa Fluor 647 AffiniPure F(ab')_2_ fragment donkey anti-rabbit IgG (H+L), Jackson ImmunoResearch, 711-606-152, lot #128806, RRID:AB_2340625; Alexa Fluor 488 AffiniPure F(ab')_2_ fragment donkey anti-chicken IgY (IgG; H+L), Jackson ImmunoResearch, 703-546-155, lot #127495, RRID:AB_2340375] diluted in 2° antibody buffer containing 97% PTwH0.5, and 3% NDS (37°C, 7 d). We started with a 1:500 dilution of antibody on day 1 and supplemented with four equal booster doses of 2° antibody (4 × 1 d) for a final dilution of 1:100 on day 5. After one week of 2° antibody treatment (five additions over 5 d plus two extra days), we performed washes in PTwH0.5 over 4 d (37°C, 4 × 8–12 h followed by 2 × 1 d).

### Clearing

Initial clearing steps were performed in 15-ml conical polystyrene centrifuge tubes while gently mixing on the rotating mixer, and sample tubes were filled to the top with solutions to prevent oxidation. We first dehydrated samples using ascending concentrations of MeOH in dH_2_O, 0%, 20%, 40%, 60%, 80%, 100%, 100% MeOH (RT, 1.5 h each). We then performed delipidation using 66% DCM + 33% MeOH (RT, 2 × 3 h, followed by 1 × overnight). Next, we washed the samples in 100% DCM (RT, 2 × 3 h) to remove MeOH and then incubated them in dibenzyl ether (DBE) for clearing and refractive index matching (RT, 2 × 3 h, followed by 1 × overnight).

### Light-sheet fluorescence microscopy (LSFM) imaging

We used a light sheet microscope (UltraMicroscope II, LaVision Biotec) with an attached camera (Andor Neo sCMOS) and a 2×/0.5 NA objective (MV PLAPO 2XC, Olympus) with noncorrected dipping cap. Cleared tissue was imaged coronally (olfactory bulb side up) using a customized sample platform. We took images in the 488-nm (autofluorescence or Thy1-GFP signal) and 647-nm (Fos) channels and used a z-step size of 2.5 μm. We used Imspector Microscope software (v1.44) to control image acquisition with the following parameters: exposure = ∼100 ms, sheet NA = 0.156 (5 μm), sheet width = 80%, zoom = 0.63×, dynamic horizontal focus = 7, dynamic horizontal focus processing = blend, merge light-sheet = blend.

### Image preprocessing

We used the 3D rendering software Arivis Vision 4D (3.0.0) to qualitatively check for skew in the coronal alignment in our imaging dataset. We manually corrected alignment to the coronal plane using the software’s Data Transformation Gallery and exported the images as TIFF files.

### SMART R package development

We wrote all package functions in base R (R Core Team, 2019). We wrote package documentation with roxygen2 (Wickham et al., 2018). We used the magick package (Ooms, 2018) to easily load, save, and modify images; to track function durations, we used the tictoc package (Izrailev, 2014). We used the rgl (Adler et al., 2019), misc3D (Feng and Tierney, 2013), and sunburstR (Bostoc et al., 2019) packages to generate 3D interactive plots, while we used ggplot2 to generate morph plots along the AP axis. We used d3r (Bostock et al., 2019) to easily convert data frames in R into JSON hierarchies. We included these packages, along with WholeBrain (Fürth et al., 2018) as dependencies in SMART. We also include a dependency on the devtools package (Wickham et al., 2019) to facilitate easy installation from GitHub. Finally, we used the Shiny package (Chang et al., 2018) to create an interactive web applet to display our example dataset (https://smartrpackage.shinyapps.io/smart_sample_dataset).

### Package installation and Docker image

A list of online resources for installation and use of SMART and its dependencies are provided in Extended Data Table 1-1. We include a static docker image so new users can test the core features of SMART and its applicability to their data. We recommend that long-term users and those with large datasets follow the online instructions provided to install WholeBrain and SMART natively on their computers within an R development environment (e.g., RStudio).

10.1523/ENEURO.0482-21.2022.t1-1 Extended Data Table 1-1 A list of the online video tutorials available for different aspects of the pipeline. Detailed descriptions of each tutorial component are listed. Download Table 1-1, DOCX file.

### Data storage and user reference material

Our compressed raw example image dataset is provided online at https://osf.io/y9uax/, courtesy of The Open Science Framework, a free online project management repository. It is easily extractable using 7-Zip, an open-source file archiver.

## Results

### Pipeline features

**Figure 1.**
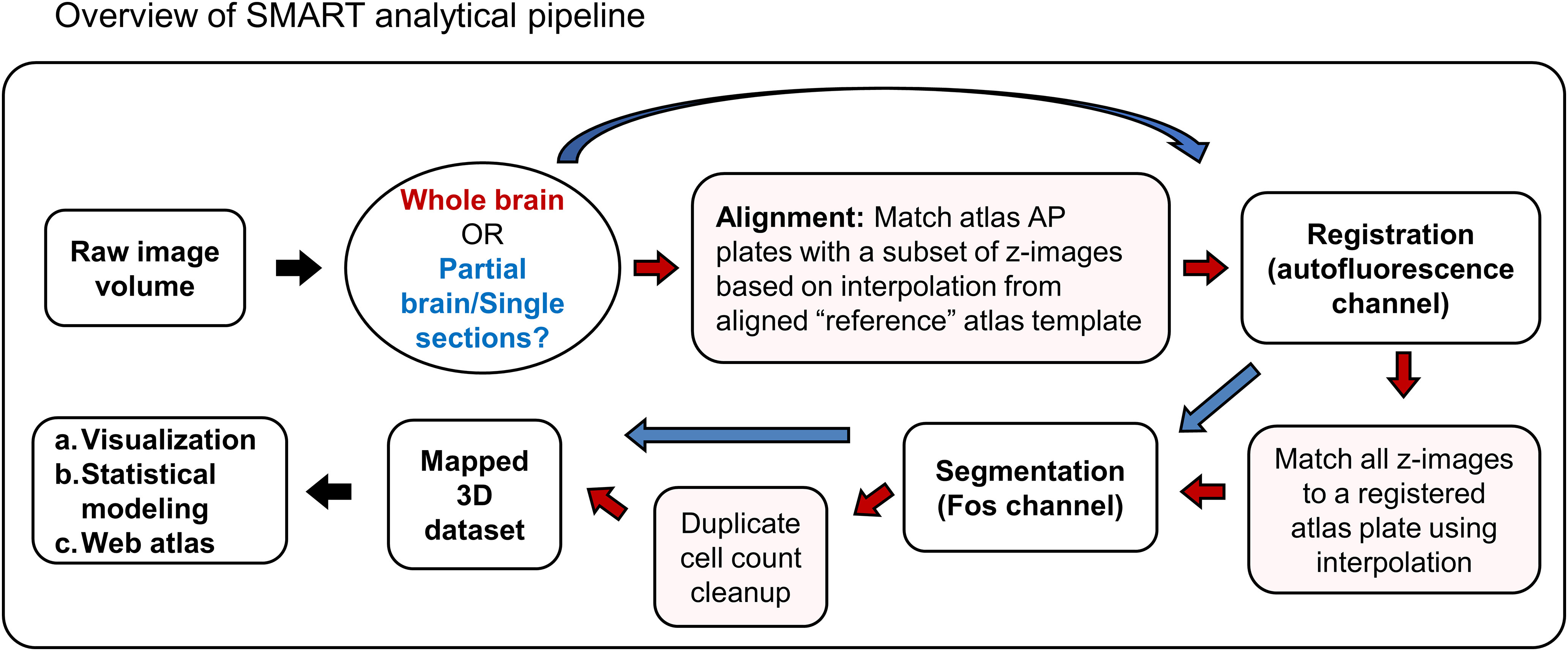
Overview of the SMART analytical pipeline. Red arrows indicate the appropriate trajectory of an intact brain imaging dataset through steps in the pipeline, while blue arrows indicate the appropriate trajectory for a partial brain dataset, consisting of coronal sections chosen by the user. Video tutorials for each step outlined in the schematic are provided in Extended Data Table 1-1.

SMART builds on the WholeBrain package to process intact whole and partial brain datasets (Fig. 1*A*). Key improvements include: (1) extensive video tutorials (Extended Data Table 1-1) and written documentation to support SMART package installation and implementation; (2) a unified setup function to define parameters before registration, segmentation, and anatomic assignment (forward warp); (3) a user-friendly console interface during registration for easy addition, modification, and removal of correspondence points or reversion to the previous modification; (4) nonuniform AP deformation is accounted for using semi-manual alignment to user-defined “reference” atlas templates; (5) analysis steps are looped through the entire image dataset with simple function calls; (6) duplicate cell counts assigned to adjacent z-planes during 2D segmentation are identified and removed, extending WholeBrain segmentation to 3D volumes; (7) data output at each step is saved and organized in a standardized format that allows modifications without restarting the pipeline; (8) additional SMART functions provide new ways to parse and visualize data across ROIs. A comprehensive list of all SMART functions is provided in the package documentation and detailed in the online tutorial. We highlight some key features in depth in the remainder of this section.

### Software installation

Installation of the SMART package is simple, and accomplished with a single command using the devtools package in R. However, since WholeBrain is a SMART package dependency, it must be preinstalled. We include links to up-to-date instructions for WholeBrain installation on Linux, Windows, and Mac-based operating systems on the package website (https://sgoldenlab.github.io/SMART/index.html). Additionally, to facilitate quicker access to the pipeline to trial WholeBrain and SMART functions, we used Docker (Merkel, 2014), a platform for software virtualization, and created a Linux-based static docker image with WholeBrain and SMART prepackaged. The website also includes instructions to download and run a container of our docker image, a video tutorial of the installation, and our recommendations regarding its usage.

### Setup

Analysis parameters are stored in a single variable list created by running the interactive function, setup_pl(). All stored parameters are easily modifiable, and this variable list is the main input necessary for all downstream analysis. The function im_sort() orders image paths based on user-defined, flexible, microscope file naming conventions, and the function get_savepaths() generates standardized subdirectories for analysis outputs.

### AP alignment

**Figure 2.**
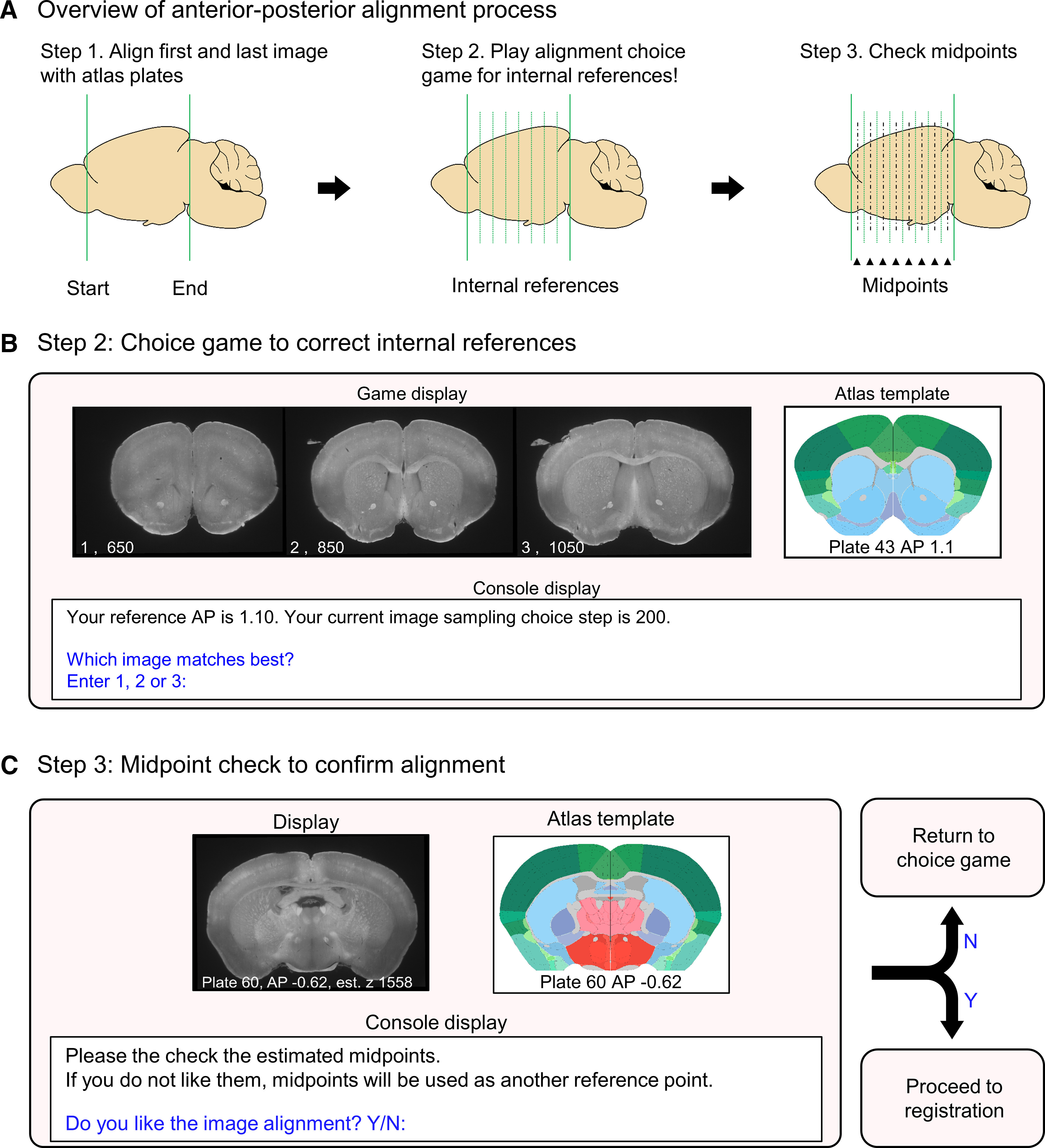
Diagram of the alignment process to reference templates. ***A***, A schematic illustrating the process of qualitative alignment and inspection of midpoints of reference templates. ***B***, A visual representation of the graphical windows displayed during the choice game and the user options allowed in the R console window. The choice game is cycled through each internal reference template. During the midpoint check, the choice game is automatically played again for midpoints that are unsatisfactorily aligned; these midpoints become additional reference templates. ***C***, A visual representation of the graphical windows displayed during the qualitative midpoint check and the user options in the R console.

SMART corrects nonlinear relationships between the reference atlas and imaged dataset in the AP axis by interpolating between a subset of user-aligned references plates (Fig. 2*A*). First, the start and end images in the dataset are manually assigned AP coordinates by comparing against a standard atlas. We provide an example atlas (accessible through the tutorial website) based on the Allen Mouse Common Coordinate Framework plates used in the WholeBrain package. Next, users specify the AP coordinates of internal reference template plates with histologic landmarks that are either most familiar or most relevant to their experiment. If unspecified, they default to seven suggested plates (+1.91, +1.10, −0.42, −0.93, −1.94, −2.95, −3.96 mm bregma) across the AP axis. Table 3 provides descriptions of suggested anatomic landmarks as a guide for users to identify these default coordinate plates.

**Table 3:** List of the seven default coordinates used in the “choice game” to align experimental whole-brain mouse LSFM images to the Allen Mouse Brain Atlas (AMBA)

AP coordinate	Plate #	Anatomical landmarks and characteristics
+1.91	35	(1) The folds separating the piriform area (PIR) from the orbital area (ORB) span across approximately half the horizontal length of a hemisphere. (2) There is a distinct teardrop shape to the anterior commissure, olfactory limb (aco). (3) The nucleus accumbens (ACB) has not yet appeared.
+1.10	43	(1) The two “wings” of the corpus callosum, anterior forceps (fa) are just about to touch. (2) The diagonal band nucleus (NDB) is clearly present and extends halfway the length from the ventral-most point of the midline to the corpus callosum.
−0.42	58	(1) The columns of the fornix (fx) and the stria medullaris (sm) have just separated; the stria medullaris is still connected in one piece. (2) The optic chiasm (och) is directly below the hypothalamus in one piece and has not yet split into two optic tracts (opt).
−0.93	63	(1) The CA3 stratum oriens (CA3so) of the hippocampus is just beginning to show within the fimbria (fi); however, multiple layers of the CA3 are not showing yet. (2) The columns of the fornix (fx) have migrated below the top of the third ventricle. (3) The optic tracts have migrated close to the border of the hypothalamus and amygdala.
−1.94	73	(1) The dentate gyrus (DG) is clearly present and spans approximately 2/3 the horizontal length of one wing of the hippocampus. (2) The ventral-most point of the granule layer of the dentate gyrus (DG-sg) is roughly the same height as the ventral-most point of the pyramidal layer of CA3 (CA3sp). (3) The fasciculus retroflexus (fr), mammillothalamic tract (mtt), and columns of the fornix (fx) are clearly seen in their appropriate positions and spaced evenly apart; the mtt is slightly closer to the fx than the fr. (4) The median eminence is (ME) is clearly visible at the bottom of the midline.
−2.95	83	(1) The medial mamillary nucleus (MM) of the hypothalamus has receded and is connected by a thinned layer of hypothalamic tissue. (2) the posterior commissure (pc) and cerebral aqueduct have migrated down from the 3rd ventricle (V3), and they are separated from V3 by sublayers c and b (SCig-c, S-Cig-b) of the superior colliculus.
−3.96	93	(1) The tegmental reticular nucleus (TRN) and pontine gray (PG) are easily identified and separated by the corticospinal tract (cst) and medial lemniscus (ml). (2) The brachium of the inferior colliculus (bic) protrudes out to the left and right of the midbrain, slightly beyond the middle cerebral peduncle (mcp).

Detailed descriptions of the anatomic landmarks used to match experimental images with the plates are listed. Region acronyms used in the AMBA are also provided so users can cross validate with the AMBA (http://atlas.brain-map.org/atlas?atlas=1).

The selected internal reference templates are aligned to the imaging data through an interactive “choice game” where three image options are presented alongside the reference atlas template (Fig. 2*B*). The center image is estimated based on interpolation of the first and last aligned image, while the left and right images are further anterior and posterior options, respectively. The choice presentations become progressively closer to each other when the user chooses the middle image, while a choice of the right or left image sets them as the new middle image during the next choice cycle. The default progression of z-step choices between consecutively presented images for each coordinate is 200, 100, 30, and 10 images, although this progression is user-modifiable. Once all internal reference templates have been aligned, users can run a midpoint check (Fig. 2*C*) to confirm alignment and play the choice game again if needed. All intermediate AP coordinates are estimated by interpolating between reference templates.

**Figure 3.**
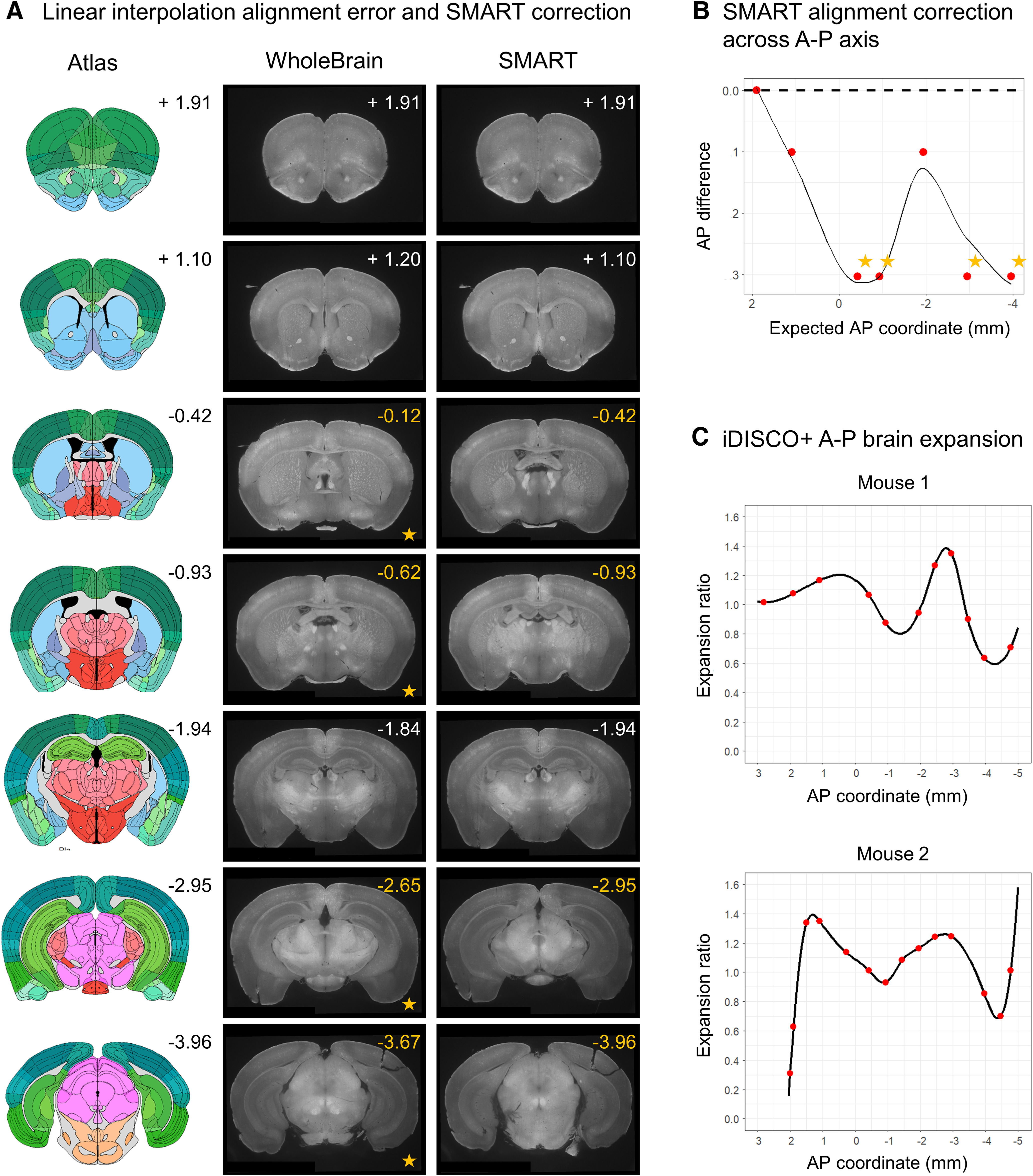
Comparison of linearly interpolated versus choice game-aligned images to reference templates. ***A***, A qualitative panel showing seven default internal atlas templates with their corresponding AP coordinates (left), predicted images based on linear interpolation (middle, WholeBrain), and aligned images following user alignment with the choice game (right, SMART). The actual coordinates of the images following the choice game are printed in the top-right corner of the images. ***B***, A plot of the difference between the reference coordinates and the actual AP coordinates of the images based predicated based off linear interpolation. Starred are four AP coordinates with the greatest absolute difference; they correspond to the starred images in ***A*** for qualitive comparison. ***C***, Normalized brain morph ratio across the AP axis for two example datasets following the choice game and a midpoint-check. Red dots indicate positions of the aligned reference templates, and the black line indicates interpolated morph ratio for AP positions between aligned reference templates.

We demonstrate the utility of the choice game by comparing the difference between predicted AP coordinates of images based on simple linear interpolation, a strategy used in base WholeBrain, and their actual coordinates following the choice game (Fig. 3*A*). Choice of images that aligned accurately to the reference plates were based on detailed inspection of the anatomic landmarks listed in Table 3. Several images differed as much as 300 μm from their predicted AP values, enough to cause significant errors in assignment of detected Fos-positive cell counts to the correct anatomic regions (Fig. 3*B*). We provide a plotting function after users play the choice game to show the extent of nonlinear deformation in iDISCO+ processed brain samples along the AP axis relative to the Allen Mouse Brain Atlas (Fig. 3*C*).

### Interactive registration improvement

**Figure 4.**
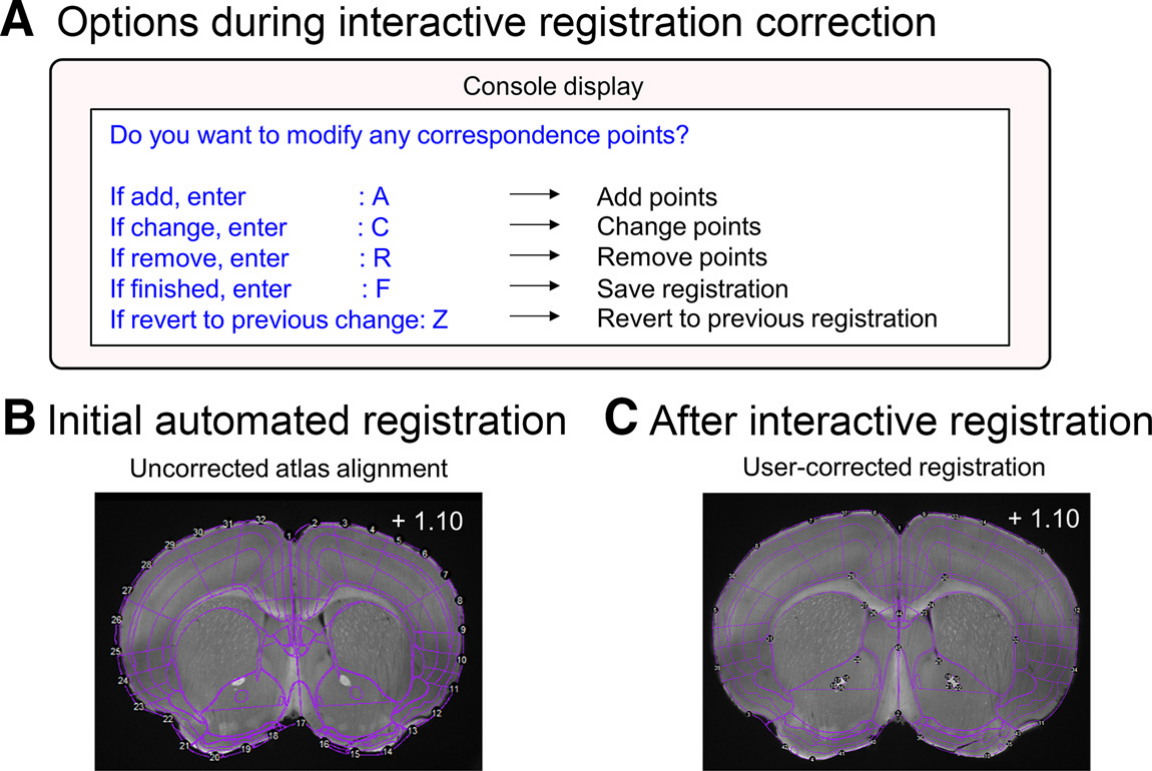
The interactive registration correction process. ***A***, A visual representation of user options in the R console display during manual correction of registrations. ***B***, An example image showing the initial registration of the atlas template to the tissue based on correspondence points around the contours of the tissue in the autofluorescence channel. ***C***, The atlas-tissue alignment is improved following interactive registration correction.

In the base WholeBrain package, removing, changing, or adding registration correspondence points can be tricky for new users as each change requires a separate function call, and it is not possible to revert to a previous state. When registering large volumes with small image z-steps, this is prohibitive. SMART incorporates all steps into a single registration function, regi_loop() that provides an interactive console interface, allows reverting to the last modification, automatically loops through selected atlas registration plates (Fig. 4*A*), and saves the output of each successful atlas registration (Fig. 4*B*).

### Automated segmentation, duplicate cell count cleanup, and forward warping

The features of interest segmented by WholeBrain are defined by parameters assigned to a segmentation filter. Using the filter as an additional input, SMART automatically loops through the entire image dataset and segments according to the filter parameters. For datasets with a high-resolution z-step, a major issue is the segmentation of the same cell across multiple adjacent images. The SMART function clean_duplicates() corrects for inaccurate artificially inflated cell counts with the dataset based on user-defined distance thresholds in the *z*-axis and the *x-y*-axes. The central position of the cell body is marked in the *z*-position containing the maximum intensity values while duplicate counts across other images are erased. The function forward_warp() loops the WholeBrain warping function through the entire dataset and assigns anatomic *x-y-z*-positions to corrected segmentation data.

### Pipeline validation with intact cleared brain LSFM dataset

**Figure 5.**
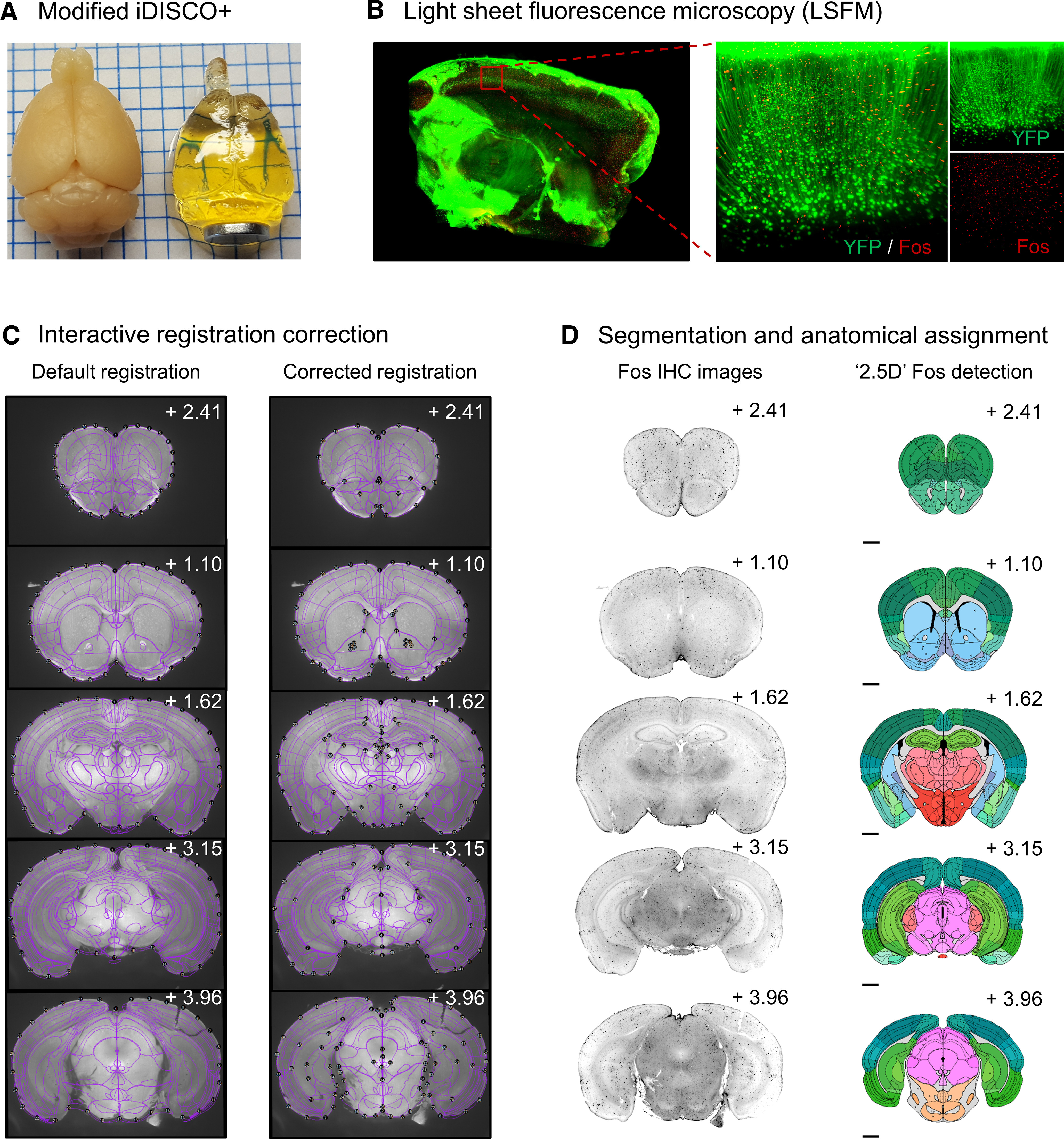
Representative images of registration correction and Fos segmentation from an example dataset. ***A***, Transparency of intact mouse brain samples pre-iDISCO+ and post-iDISCO+ immunolabeling and clearing. ***B***, Example LSFM tissue section of an intact cleared brain from a Thy-1 GFP transgenic mouse (left); an enlarged cortical image in the YFP and Fos imaging channels (right). ***C***, Representative images of initial and corrected atlas-tissue registrations in the autofluorescence channel (488 nm) of the example dataset from Mouse 1 at various AP coordinates. Note the improved alignment of internal structures such as ventricles and white matter tracts. ***D***, Images of Fos-IHC (647 nm) and segmented Fos-positive cells following automated 2.5D segmentation, cleanup of duplicate cell counts and forward warping onto atlas space.

We first validated our modified iDISCO+ clearing approach (Fig. 5*A*) and immunolabeled the IEG Fos throughout the intact tissue of a Thy-1 GFP transgenic mouse (Fig. 5*B*). We then cleared and immunolabeled Fos in male outbred CD-1 mice (Fig. 5*E*, Mouse 1). We used SMART to correct AP deformation (choice game), manually correct registration misalignments in the autofluorescence channel (488 nm) across all atlas plates (Fig. 5*C*). We then segmented cell bodies in the Fos channel (647 nm), corrected for duplicate segmentation objects, and performed the forward warp of corrected counts onto atlas space (Fig. 5*D*).

### Data organization and visualization

**Figure 6.**
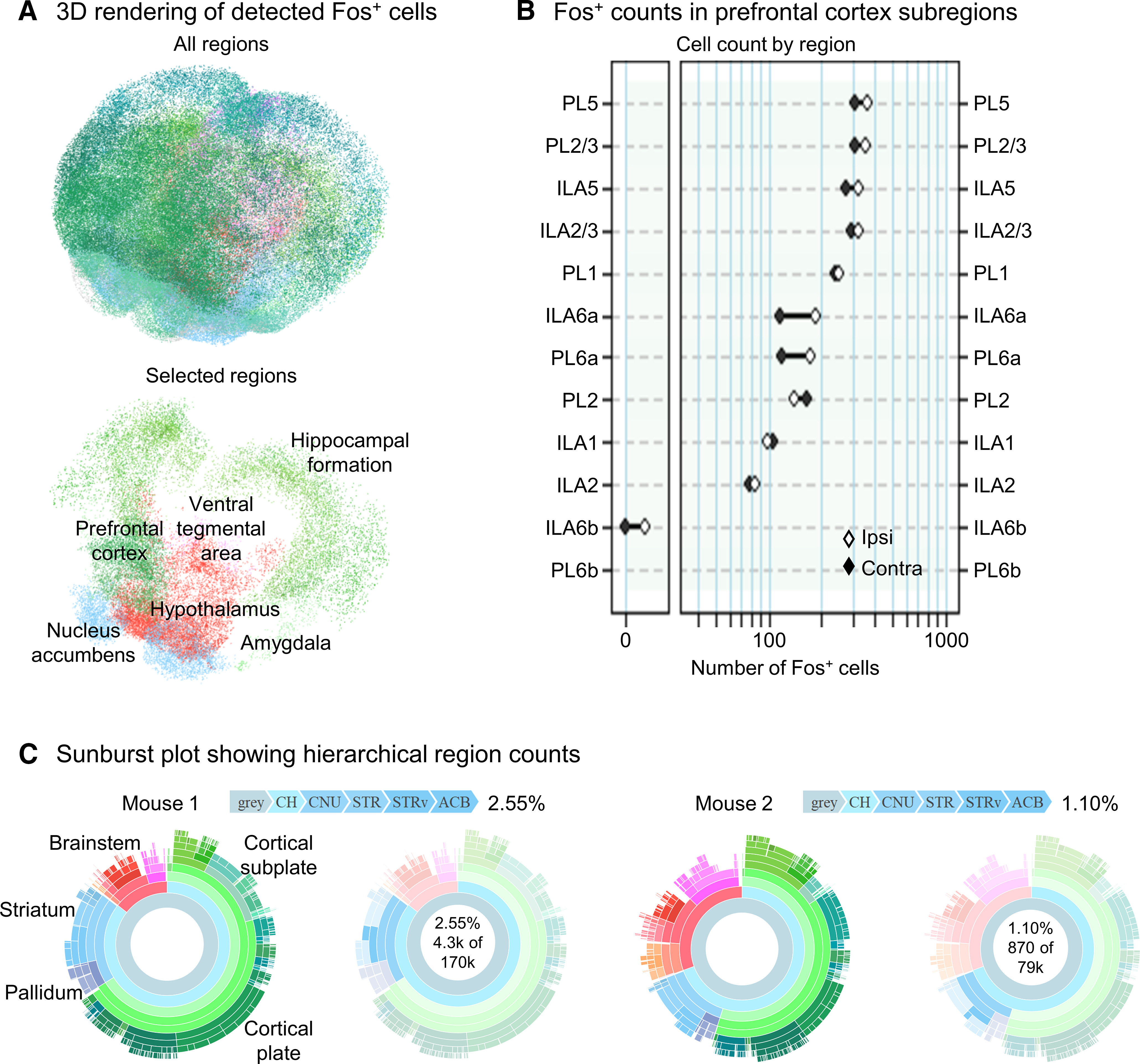
Graphical representations of whole-brain Fos datasets following SMART registration, and segmentation. ***A***, 3D rendering of the entire mapped dataset (Mouse 1) from Figure 4, left, and a 3D rendering of the indicated ROIs (right). ***B***, Region plot quantifying segmented Fos-positive cell bodies in the PFC (Mouse 1, PL and IL). ***C***, Sunburst plots showing region cell counts and hierarchical structural relationships for two example datasets. The innermost ring represents all cell counts, with each subsequent outer ring representing child structures of the adjacent inner ring. Regions colors are based on the Allen Mouse Brain Atlas and arc length is proportional to total cell counts. Plots are interactive and hovering over an arc reveals region identity, hierarchical path, and region cell count (right). ACB, nucleus accumbens; IL, infralimbic cortex; PL, prelimbic cortex.

SMART includes additional functions for visualization of analysis outputs. The function get_table() creates a table displaying all region acronyms, region cell counts, and region cell count percentages in the dataset, while get_rois() extracts only user-specified ROIs from the main dataset. These SMART functions can be combined with existing WholeBrain visualization functions to generate interactive 3D renderings and region cell count plots of user-specified ROIs. We demonstrate these features in our example dataset by generating a 3D rendering of various limbic regions (Fig. 6*A*) and a region cell count plot of the PFC (Fig. 6*B*). In addition to the normalized morph plot along the AP axis (Fig. 3*C*), we demonstrate an interactive sunburst plot showing region cell counts and hierarchical structural relationships as an additional data visualization feature (Fig. 6*D*). We provide an interactive web applet (https://smartrpackage.shinyapps.io/smart_sample_dataset) for sharing and 3D visualization of SMART outputs.

## Discussion

We present SMART, an open-source R package extending the functionality of the WholeBrain analytical pipeline to partial and brain-wide activity mapping using mouse LSFM datasets. Implementation of the SMART analysis pipeline does not require prior programming expertise. Users are guided through the analysis setup process and presented with an intuitive console interface where needed. The modified registration, segmentation, and forward warp functions automatically loop through the entire imaging dataset, a valuable feature when analyzing large neuroimaging datasets. SMART also automatically creates and saves outputs in standardized data subdirectories, facilitating organized storage and sharing of datasets. Further, SMART accounts for nonuniform morphing along the AP axis in cleared tissue datasets and corrects for possible duplication of cell counts during 2D segmentation. We also present a modified Fos immunostaining protocol for uniform labeling and demonstrate SMART’s key features using an example mouse LSFM dataset generated following this protocol. While we describe SMART’s application to a whole mouse brain LSFM Fos dataset, many of these features can be implemented on LSFM scans of partial brains or even images of single sections and are not restricted to the staining technique or image acquisition parameters used in this manuscript.

An important consideration when using the SMART pipeline is the speed of analysis. The analysis bottleneck of the base WholeBrain package is the manual correction of registered atlas plates. While this is the most time-intensive step and is less efficient than automated methods (Ni et al., 2021), it enables interactive improvement of registration based on users’ expertise. Users can save significant time registering an entire brain using SMART’s interactive console interface. Using a comparable dataset to the one presented, we estimate that a well-trained user of the SMART pipeline can accurately register an entire mouse brain following 3–4 d of dedicated registration. The remaining segmentation, duplicate cell count cleaning, and forward warping processes can be completed within 1 d. A notable feature of SMART is that it allows users to save intermediate stages of analysis and return to them when convenient. This makes it possible for multiple users to work on the same data in tandem and spread the time commitment across entire teams.

The main determinant of the accuracy of region cell count mapping is the pipeline user’s own anatomic knowledge. This remains both an advantage and disadvantage of the SMART-WholeBrain approach compared with other typically intensity-based voxel registration approaches: users can easily evaluate registration quality and correct for misalignments, but between-user reproducibility in registrations requires that users have similar anatomic expertise. However, we propose that using these approaches in parallel provides the best outcome. An initial registration pass using automated voxel-based approaches like ClearMap (Renier et al., 2016), can provide a broad view of activity patterns across the entire brain and highlight zones along the AP axis with ROIs. This can be followed up by user-guided fine-tuned registration in SMART (using coronal plates restricted to these identified AP axis limits) to produce detailed counts of individual regional subdivisions. These counts can serve both as an orthogonal cross-validation of ClearMap outputs and allow further isolation of target region boundaries in the AP axis. Additionally, the results are easily compared with prior classical slice section-based IEG studies, and provide refined stereotaxic targets for resulting chemogenetic, optogenetic, and pharmacological approaches to test for a functional role of any detected target regions. SMART outputs may also be visualized and shared through dedicated brain visualization software such as BrainRender (Claudi et al., 2021).

Mapping of cell distributions across intact brain tissue is becoming an integral approach toward understanding the activity and function of neural circuits at the level of the connectome. The resurgence in interest in tissue clearing techniques within the last decade has driven new advancements in high resolution volumetric imaging methodologies, such as serial two-photon tomography (Ragan et al., 2012) and LSFM (Dodt et al., 2007). Such methods faithfully capture biological information at sub-cellular resolution, but in doing so, they generate unwieldy sizes in neuroimaging datasets. A key bottleneck is the registration and cellular segmentation of these massive datasets to a standardized atlas to enable across-subject and across-experimental group comparisons.

While automated voxel-based registration approaches provide a high-throughput solution, they typically have high computational demands, require programming expertise, and rarely allow for user-evaluation or manual correction. Presently, the most common voxel-based approach is the use of ClearMap (Renier et al., 2016; Kirst et al., 2020), recently updated to ClearMap2. In contrast, WholeBrain’s spline-based computational framework is versatile because of its minimal processing requirements, scale-invariant registration, and manual registration correction capabilities. To accommodate these technical differences, minimum processing requirements between these are approaches are large: ClearMap requires high-level multi-CPU processing and minimum of 256 GB RAM while WholeBrain accommodates standard CPU processing and a minimum of 32 GB RAM. Other technical differences include the level of registration automation, where ClearMap is fully automated and WholeBrain is semi-automated, and programming environment, where ClearMap is written in Python and WholeBrain in R. These considerations may help users select the more appropriate approach for analysis of their datasets. However, the WholeBrain package was primarily built to analyze serial sections across the AP axis with wide z-spacing and is not optimized for analysis of high z-resolution LSFM datasets.

SMART extends WholeBrain’s functionality in this domain, and streamlines the user experience through installation, setup, registration, segmentation and analysis. SMART lowers the barrier to entry by guiding users through each individual step of installation and analysis, includes qualitative checks during each step of the process, and provides simple tools for visualization of results. It retains the core strengths of WholeBrain (e.g., low processing requirements, user-guided registration correction) and adds new functions to support analysis and visualization of partial and whole mouse brain LSFM datasets.

We expect the SMART package will enhance the accessibility of the base WholeBrain package and encourage more users to undertake neural mapping projects, whether using full brain or partial brain datasets. To this end, we include a detailed website to guide new users through installation of the necessary software and package dependencies and provide step-by-step instructions and video tutorials (Extended Data Table 1-1) of SMART’s key functions using a downloadable example dataset. We also include a docker image installation option with WholeBrain and SMART prepackaged together for easier accessibility. We also provide our pipeline output as a convenient web applet for interactive visualization. The package website and description are available at https://sgoldenlab.github.io/SMART/ and also as the Extended Data 1.

10.1523/ENEURO.0482-21.2022.ed1 Extended Data 1 SMART code. Download Extended Data 1, ZIP file.

## Synthesis

Reviewing Editor: Christie Fowler, University of California, Irvine Decisions are customarily a result of the Reviewing Editor and the peer reviewers coming together and discussing their recommendations until a consensus is reached. When revisions are invited, a fact-based synthesis statement explaining their decision and outlining what is needed to prepare a revision will be listed below. The following reviewer(s) agreed to reveal their identity: Kevin Beier.

This manuscript was reviewed by two experts in the field. Both reviewers felt that the manuscript provided valuable information for the field with the ‘SMART’ open package for R for whole brain analysis of cleared tissue. It was noted that, if user-friendly, this would be a significant advance over currently available software packages. However, the following concerns were noted:

1. The major concern was that the software installation was not easy. One reviewer spent at least 4 dedicated hours trying to get the prerequisite WholeBrain to work on both windows and ubuntu machines, without success, and there wasn’t a clear path forward. As the majority of open issues (14/24) on the Github for WholeBrain related to difficulty in installation, perhaps this is a common problem.

2. It was also noted that the manuscript would be strengthened by the authors demonstrating how the alignment of samples in WholeBrain is inadequate and show how SMART improves it. And/or provide cell counts of the cFos-cleared brains and alignments with different regions and make the case that SMART improves the quality/variability of analysis. Video tutorials may also help. Anything that allows the readers to assess how actually useful their package is without more validation.

3. It was felt that a more thorough technical comparison to ClearMap would be uselful. WholeBrain tends to be in less wide use than ClearMap, and while it has its advantages, it would also be useful to provide a comparison of how well each of the packages works for data analysis. Some benefits are mentioned in the manuscript (e.g., ability to map incomplete brains onto a reference space), but a more comprehensive comparison would help readers to better understand the benefits of their approach over others that are available.

## Author Response

Synthesis Statement for Author (Required): This manuscript was reviewed by two experts in the field. Both reviewers felt that the manuscript provided valuable information for the field with the ‘SMART’ open package for R for whole brain analysis of cleared tissue. It was noted that, if user-friendly, this would be a significant advance over currently available software packages. However, the following concerns were noted: We appreciate the editors and reviewers’ positive reception. Because of the double-blind formatting requirements for the initial submission, we were asked to remove documentation that would unblind the review process, including written and video tutorials. We now include these in our revision. We take the general comment on user-friendliness seriously and have spent considerable effort to increase user-friendliness and accessibility of SMART for future users. These are detailed in the specific responses to reviewer comments below, but are summarized by: 1. We have improved WholeBrain installation documentation within the manuscript (p. 8-9) and on the SMART github page (https://sgoldenlab.github.io/SMART/installation.html). 2. To facilitate quicker access to the SMART pipeline to trial SMART functions, we used Docker, a platform for software virtualization, and created a Linux-based docker image with WholeBrain and SMART pre-packaged. This provides a rapid and easy, albeit static, WholeBrain and SMART installation. 3. We have generated seven detailed video tutorials spanning WholeBrain and SMART installation through final data export and plotting (see new Supplemental Table 1). 1 1. The major concern was that the software installation was not easy. One reviewer spent at least 4 dedicated hours trying to get the prerequisite WholeBrain to work on both windows and ubuntu machines, without success, and there wasn’t a clear path forward. As the majority of open issues (14/24) on the Github for WholeBrain related to difficulty in installation, perhaps this is a common problem. We appreciate the reviewer’s comment on the difficulty associated with the installation of WholeBrain and acknowledge this is a common problem for WholeBrain users. In this revision, we have approached this from two directions. First, we have improved our written installation instructions for WholeBrain in the SMART installation page (https://sgoldenlab.github.io/SMART/installation.html). Second, we now provide a Docker image with the WholeBrain and SMART packages pre-installed as a Docker container, coupled with both written instructions as well as a detailed video tutorial (Supplemental Table 1, Video 1). We detail these installation options in newly added text (p. 8-9) in the revision. This alternative installation pipeline is recommended for those who are having serious installation issues. The docker can also be used to trial the functions that are available in SMART. Because the docker image is a “snapshot” of the current versions of both WholeBrain and SMART at the time of the image creation, any updates to these packages will not be included in the image. Further, Docker requires X-Forwarding to render certain graphical user interfaces onto the host computer. This results in a slower overall interactive experience. Therefore, we still suggest users attempt to install WholeBrain normally, if possible. However, it should now be notably easier for users to trial the use of SMART prior to dedicating effort to a full manual installation. If needed, they can also use SMART (and WholeBrain) as normal with this installation approach, although without easy updates to either package and slower GUI experience. 2. It was also noted that the manuscript would be strengthened by the authors demonstrating how the alignment of samples in WholeBrain is inadequate and show how SMART improves it. And/or provide cell counts of the cFos-cleared brains and alignments with different regions and make the case that SMART improves the quality/variability of analysis. Video tutorials may also help. Anything that allows the readers to assess how actually useful their package is without more validation. We appreciate the reviewers’ thoughtful comments on demonstrating the improved sample alignment. In this revision, we demonstrate the efficacy of the SMART “choice game” by comparing the difference between AP coordinates of images predicted to correspond to the default reference templates based on simple linear interpolation, the strategy used in base WholeBrain, and their actual coordinates following the choice game (new Fig. 3A). There were several images that differed as much as 300 microns from their predicted values, enough to cause significant difference in qualitative comparisons (Fig. 3B). We now include this direct comparison in the revised manuscript (p. 10, and new Figure 3). Further, we now also include our video tutorials for users. These include: (1) WholeBrain-SMART docker installation, (2) Pipeline Introduction, (3) Pipeline Setup, (4) Alignments using the Choice Game, (5) Interactive Registrations, (6) Segmentation and Forward Warping, and (7) Dataset manipulation and plotting (see new Supplemental Table 1 for links to videos). Combined with the new Docker-based installation option, we hope these video tutorials and simpler installation process with allow readers to better assess the utility of the SMART package for their research. 2 3. It was felt that a more thorough technical comparison to ClearMap would be uselful. WholeBrain tends to be in less wide use than ClearMap, and while it has its advantages, it would also be useful to provide a comparison of how well each of the packages works for data analysis. Some benefits are mentioned in the manuscript (e.g., ability to map incomplete brains onto a reference space), but a more comprehensive comparison would help readers to better understand the benefits of their approach over others that are available. We appreciate the reviewers’ thoughtful comments on improving our discussion of the technical comparison between 3D voxel-based pipelines like Clearmap and 2D spline-based packages like WholeBrain-SMART. We now include a more detailed technical comparison in the discussion of the resubmitted manuscript (p. 13-14). Paired with the detailed written github and video tutorials, we hope it will now be easier for potential users to identify if the SMART pipeline is appropriate for their research needs. Further paired with the Docker installation option, they can also more rapidly pilot their own data, before committing further time and resources or moving to an alternative analytical pipeline.
