# Supplementary material for: SMART: An Open-Source Extension of WholeBrain for Intact Mouse Brain Registration and Segmentation
Source: eNeuro. 2022 May 3;9(3):ENEURO.0482-21.2022. doi: 10.1523/ENEURO.0482-21.2022 (PMC9070730; doi:10.1523/ENEURO.0482-21.2022)
Supplement: Extended Data Table 1-1 — A list of the online video tutorials available for different aspects of the pipeline. Detailed descriptions of each tutorial component are listed. Download Table 1-1, DOCX file. [file enu-eN-OTM-0482-21-s02.docx]

**Table 1-1**. A list of the online video tutorials available for different aspects of the pipeline. Detailed descriptions of each tutorial component are listed.

| **Title** | **Description** | **Video link** |
| --- | --- | --- |
| WholeBrain-SMART docker installation | A tutorial on how to download and run a Docker image with the WholeBrain and SMART packages pre-installed as a Docker container. | <https://youtu.be/KeUzFX6B7uo> |
| Pipeline Introduction | An introduction to the pipeline schematic and the improvements that SMART builds on top of the base WholeBrain package. | <https://youtu.be/9ifnjeESgvg> |
| Pipeline setup | A tutorial on how to interactively enter the pipeline parameters in a setup list type in R. | <https://youtu.be/y_9z5Na6rUM> |
| Alignment using the choice game | A walkthrough of the choice game through the default internal reference points in an example mouse dataset. | <https://youtu.be/_dyjj9HjAkA> |
| Interactive registrations | A demonstration of the regi_loop() function to interactively improve the registration quality of the aligned atlas plate using a console interface. | [https://youtu.be/hC0HLP63E CM](https://youtu.be/hC0HLP63E%20CM) |
| Segmentation and forward warping | A demonstration of the seg_loop(), clean_duplicates(), and forward_warp() functions which automatically loop through an example dataset to count cells, clean duplicates cell counts, and warps the cell counts onto atlas space. | <https://youtu.be/A8KgfdGzyrE> |
| Dataset manipulation and plotting | A demonstration of the different data parsing functions, as well as various interactive visualization functions that SMART extends from WholeBrain. | <https://youtu.be/u9w-VoponCs> |
