# Supplementary material for: SMART: An Open-Source Extension of WholeBrain for Intact Mouse Brain Registration and Segmentation
Source: eNeuro. 2022 May 3;9(3):ENEURO.0482-21.2022. doi: 10.1523/ENEURO.0482-21.2022 (PMC9070730; doi:10.1523/ENEURO.0482-21.2022)
Supplement: Extended Data 1 — SMART code. Download Extended Data 1, ZIP file. [file enu-eN-OTM-0482-21-s03.zip › SMART/README.html]

# SMART: Semi-manual alignment to reference templates

---

Author(s): Maintainer: redacted for review - redacted for review

Contributor: redacted for review - redacted for review

### Introduction:

This package builds a pipeline interfacing with the wholebrain package [1] to process whole brain imaging datasets. Datasets of a handful of single slice images across the brain can also be fed into this pipeline. For brevity, I’ll refer to these datasets as partial brains.

There are five main advancements that SMART builds on top of the wholebrain base package:

1. Guides the user through setting up analysis parameters and feeds the imaging data through registration, segmentation, and forward warp process. It automates looping through these analyses with simple function calls so the user does not have to set up this looping.
2. Provides a user-friendly interface whenever possible. This includes a consoles changing, and removing correspondence points or reverting back to a previous correspondence change.
3. Helps to solve non-linear relationships between atlas AP coordinates to actual distances in the anterior-posterior axis in real imaging datasets. This is critical when dealing with tissue clearing methods that cause non-uniform tissue morphing across the AP-axis.
4. Organizes and stores the output of wholebrain analysis in automatically generated subdirectories. This standardized organization will be beneficial when sharing data output from wholebrain.
5. Provides more ways to parse whole brain datasets and visualize data across ROIs.

*Disclaimer. This package is intended for coronal plane datasets. Preprocessing must be done to align the dataset coronally before it should be analyzed with the pipeline.*

**Below is an illustration of the pipeline schematic:**

---

### Installation of SMART:

To use SMART, wholebrain must first be installed. The instructions to install wholebrain can be found here. Please note, if you are using a windows machine, manual installation of opencv is no longer necessary. I highly recommend reading through some of the documentation for wholebrain before using this package.

SMART is easily installed from github using the devtools package:

```
# Load devtools
library(devtools) 
 
# Install SMART
devtools::install_github("redacted for review/SMART")

# Load SMART
library(SMART)

# Pull up package descriptions and list of functions
?SMART
```

---

### Pipeline breakdown:

As illustrated in the schematic and stated in the package descriptions, not all parts of this pipeline will be used when analyzing a partial brain. Sections/functions exclusively meant to be used with whole brain datasets will be marked with **(W)**. Additionally, some package functions may be useful for analysis outside the pipeline we present here.

For processing a wholebrain dataset, the pipeline is split into 5 sections. These sections are listed below along with the functions that belong to each section:

**Part 1: Setup pipeline**

- `setup_pl()` User friendly way to setup parameters for whole or partial brain pipeline analysis.
- `im_sort()` A function to sort image paths for imaging datasets.
- `get_savepaths()` Generate savepaths and save directories.

**Part 2: Alignment (W)**

- `choice()` **(W)** User friendly choice game to align internal reference atlas plates.
- `brainmorph()` **(W)** Generate a brain morph plot.
- `interpolate()`**(W)** Interpolate all AP and z numbers for atlas plates in a whole brain project

**Part 3: Registration**

- `registration2()` Console user interface built on top of registration() function from the wholebrain package.
- `regi_loop()` Automatically loops through the image registration process for the imaging dataset.

**Part 4: Segmentation, duplicate cleanup, & forward warping**

- `filter_loop()` Loops through reference slices and allows user to check/change filter settings for segmentation or registration.
- `seg_loop()` Loop through and segments images in the segmentation channel.
- `clean_duplicates()` **(W)** Duplicate cell count clean up of segmentation output.
- `forward_warp()` Performs forward warp loop back onto atlas space using segmentation and registration output.

**Part 5. Dataset manipulation and plotting**

- `get_rois()` Gets a subset of the forward warped dataframe of just regions of interest.
- `get_sunburst()` Generates a sunburst plot using a forward warped dataset.
- `get_tree()` Creates a dataframe of hierarchical region cell count data.
- `glassbrain2()` Generates a 3D plot of cells counts with option of removing glassbrain in the background.

Below is a walkthrough of each of these functions and the pipeline as a whole using an example whole brain dataset. Feel free to use the function links above to skip sections!

For convention, if a return value is given by a function, the recommended variable name is indicated in italics in the return section of each function’s help page. If this return value is a required argument for another function in the pipeline, the recommended variable name will also be the same name as the argument.

---

### Part 1: Setup pipeline

This sections sets up analysis parameters, sorted image paths, and generates savepaths and directories for the rest of the pipeline.

**Step 1.**

**`setup_pl()()`** This function asks the user for setup information. Based on input from the user, the function returns a list of parameters for either a whole or partial brain analysis.

```
# Scroll to the details section of the help page to see the setup parameters
?setup_pl()

# Run setup_pl, enter parameter information to the console, 
# and store the output list into a variable named setup

setup <- setup_pl()

# Check or modify your setup parameters 
setup <- setup_pl(setup)

# Note: Whenever a different user works on analyzing the same dataset,
# run the above command to change user initials. This will keep track of
# who did what.
```

> Tips: When providing folder paths, do not put quotes around the path to the console input.

For convention, sequential image numbers are z image numbers, even for a partial brain analysis. Z image number should start at 1 and end at the number of images. Image files should start indexing at 1 in the filenames to match image number.

For a whole brain analysis, the first and last atlas plates must be qualitatively aligned with the first and last z image numbers. Note that the `setup$internal_ref_z`, `setup$regi_AP`, `setup$regi_z` parameters are not user modifiable and will be NULL until the`choice()` or `interpolate()` functions are run.

Additionally, there are a few default pipeline parameters for whole brain analysis:

- Spacing between registrations (mm). DEFAULT: 0.100
- Segmentation step (integer). DEFAULT: 1.
- AP coordinates of internal reference planes. DEFAULT: 1.91, 1.10, -.42, -0.93 , -1.94 , -2.95, -3.96.

The above coordinates correspond to PFC, NAc, antHyp, start of HIP, posterior Hyp, VTA, and the PAG, respectively. These coordinates will be the atlas plates used to “calibrate” to internal z images. They’ll be used to interpolate and match the z images of remaining atlas plates. They were chosen because:

1. Based on our experience, they contain easy internal region landmarks that can be consistently identified by different users
2. They are somewhat evenly spaced throughout the brain.

However, these coordinates are user modifiable to account for user preference and varying AP ranges of imaging datasets. If you are using this pipeline for the first time, I recommend you take the default values.

The console code below shows the setup list I am using:

```
# Check setup list

> setup <- setup_pl(setup)
$`anim_ID`
[1] "1602"

$user_init
[1] "XX"

$regi_channel
[1] "D:/SMART_pilot_data/R15_1602_SG_Rein_NoTest_coronal_C02"

$seg_channel
[1] "D:/SMART_pilot_data/R15_1602_SG_Rein_NoTest_coronal_C01"

$output
[1] "D:/SMART_pilot_data/output"

$z_space
[1] 0.0025

$regi_step
[1] 0.1

$seg_step
[1] 1

$first_AP
[1] 2.917519

$first_z
[1] 1

$last_AP
[1] -4.971794

$last_z
[1] 2800

$internal_ref_AP
[1]  1.9060687  1.0969084 -0.4202672 -0.9259924 -1.9374427 -2.9488931 -3.9603435

$internal_ref_z
NULL

$regi_AP
NULL

$regi_z
NULL

Please review your setup information above: 
Do you want to change any settings: Y/N?

>
```

**Step 2.** **`im_sort()`** User friendly way to sort images from the registration channel. Asks for user input to account for flexible naming conventions for images.

```
# Sort images and store them 

# There will be user walk through for the 'separator' 
# and 'position' information necessary to sort images 
image_paths <- im_sort(setup, extension = "tif")

# Note: Setting the position argument will skip the user walkthrough. 
# e.g.
image_paths <- im_sort(setup, extension = "tif", position = 9)

# Check image_paths
image_paths
```

**Step 3.**

**`get_savepaths()`** Create standardized save directories based on the setup information from `setup_pl()`. Returns a list of savepaths to subfolders in the output folder.

```
# Create savepaths and generate data subdirectories automatically 
savepaths <- get_savepaths(setup)

# Check the output folder to see the subdirectories created!

# Show savepaths generated
savepaths

# To save the global environment at any time during an analysis session run: 
save.image(savepaths$envir_savepath)

# Going to the R_data folder in the output folder and clicking on the 
# .RData folder by date will revert the global environment back to a previous session.
# Saving everyday will prevent you from losing more than a day's worth of work.
```

> Tip: rerun the save.image() expression to update the global environment savepath every day.

Below shows my console output for my savepaths variable:

```
# Update and show savepaths

> savepaths <- get_savepaths(setup)
> savepaths
$`envir_savepath`
[1] "D:/SMART_pilot_data/output/R_data/Animal_1602_2019-01-07_XX.RData"

$out_reference_aligned
[1] "D:/SMART_pilot_data/output/Reference_aligned"

$out_RC_brain_morph
[1] "D:/SMART_pilot_data/output/RC_brain_morph"

$out_auto_registration
[1] "D:/SMART_pilot_data/output/Registrations_auto"

$out_registration
[1] "D:/SMART_pilot_data/output/Registrations_manual"

$out_registration_warps
[1] "D:/SMART_pilot_data/output/Registration_warps"

$out_segmentation_warps
[1] "D:/SMART_pilot_data/output/Segmentation_warpimages"

$out_segmentation_schem
[1] "D:/SMART_pilot_data/output/Segmentation_schematics"

>
```

---

### Part 2: Alignment **(W)**

This section is necessary for whole brain datasets only. Users first “calibrate” the reference atlas plates with their appropriate z image number by running `choice()` and playing the choice game. Based on the aligned reference plates, the user can match all remaining atlas plates with an interpolated z image using `interpolate().`

**Step 4.**

\*\*`choice()` (W) \*\* User friendly way to play the wholebrain choice game to align internal reference atlas plates. Automatically saves images of chosen aligned images in the folder specified by `savepaths$out_reference_aligned`.

Below is my initial console output when I run`choice()` with my `setup`, `savepaths`, and `image_paths` variables.

```
# Run the choice game and save the aligned 
# z reference number back into the setup list:

> setup <- choice(setup, savepaths, image_paths, filetype = "tif") # save chosen images as tifs
Play the choice game!
Your reference AP is 1.91 
Your current image sampling choice_step is  200 

Which image matches best?
Enter 1, 2 or 3:
```

**How the choice game works:**

The choice game will cycle through the internal reference atlas plates (represented below by dotted vertical lines). Three choice windows will popup for each reference AP coordinate, corresponding to choices 1,2 or 3, respectively. The user simply compares the corresponding atlas plate with the 3 choice windows and enters the best qualitative match.


The middlemost window (choice 2) is SMART’s best guess at the z image best aligned with the atlas coordinate based on the first and last aligned atlas plates (represented above by solid vertical lines).


> Note: The default positions the windows pop up on screen can be set by the `xpos` argument.

The current `choice_step` indicates how many z images the anteriormost (choice 1) or posteriormost (choice 3) images are from the middlemost image (choice 2). Entering 1, 2 or 3 into the console has three outcomes:

1. **Choice 1** The anteriormost image becomes the new choice 2 on the next choice option.
2. **Choice 2** On the next choice option, there is a smaller `choice_step` on the left and right images. Choosing this option will progressively “zoom in” on the `choice_step` options until the steps can’t get smaller. The choice game will then move on to the next atlas coordinate. By default, the smallest step is 10.
3. **Choice 3** The posteriormost image becomes the new choice 2 on the next choice option.

> Note: the `choice_step` progression is a user modifiable argument.


Once all the internal references atlas coordinates have been aligned, the `choice()` function will cycle through the midpoint AP coordinates of the aligned references as a quality check (represented above with black arrows). After comparing midpoints, any unsatisfactory midpoints become another internal reference point. The choice game is replayed for those midpoints.


Below are my internal reference AP coordinates and z numbers after running`choice()` with my setup:

```
# Check internal reference AP numbers
> setup$internal_ref_AP
 [1]  1.9060687  1.0969084 -0.4202672 -0.9259924 -1.9374427 -2.4431679 -2.9488931 -3.4546183 -3.9603435
 [10] -4.4660687

# Check matched image z numbers
> setup$internal_ref_z
 [1]  350  657 1305 1425 1813 1978 2202 2422 2541 2681
```

\*\*`brainmorph()` (W) \*\* Generate a brain morph plot showing areas of relative expansion or compression along the AP axis compared to the Allen Mouse Brain Atlas AP coodinates (normalized to 1). The reference atlas points used to generate the morph plot are plotted in red. Setting `saveplot = TRUE` will save the brain morph into the data folder designated by `savepaths$out_RC_brain_morph`

```
# Generate brainmorph. 
brainmorph(setup, savepaths, saveplot = FALSE)
```


**Step 5.**

\*\*`interpolate()` (W) \*\* This function interpolates all corresponding z and AP values for atlas plates that are not reference plates.

```
# Interpolate all remaining atlas plates and their z number
setup <- interpolate(setup)
```

Now checking the setup list will show all matched internal AP plates and z numbers:

```
> setup$regi_AP
[1]  2.91751908  2.81637405  2.71522901  2.61408397  2.51293893  2.41179389  2.31064885  2.20950382  2.10835878
[10]  2.00721374  1.90606870  1.80492366  1.70377863  1.60263359  1.50148855  1.40034351  1.29919847  1.19805344
[19]  1.09690840  0.99576336  0.89461832  0.79347328  0.69232824  0.59118321  0.49003817  0.38889313  0.28774809
[28]  0.18660305  0.08545802 -0.01568702 -0.11683206 -0.21797710 -0.31912214 -0.42026718 -0.52141221 -0.62255725
[37] -0.72370229 -0.82484733 -0.92599237 -1.02713740 -1.12828244 -1.22942748 -1.33057252 -1.43171756 -1.53286260
[46] -1.53286260 -1.63400763 -1.73515267 -1.83629771 -1.93744275 -2.03858779 -2.13973282 -2.24087786 -2.34202290
[55] -2.44316794 -2.54431298 -2.64545802 -2.74660305 -2.84774809 -2.94889313 -3.05003817 -3.15118321 -3.25232824
[64] -3.35347328 -3.45461832 -3.55576336 -3.65690840 -3.75805344 -3.85919847 -3.96034351 -4.06148855 -4.16263359
[73] -4.26377863 -4.36492366 -4.46606870 -4.56721374 -4.66835878 -4.76950382 -4.87064885 -4.97179389


> setup$regi_z
 [1]    1   36   71  106  141  176  210  245  280  315  350  388  427  465  504  542  580  619  657  700  743
[22]  787  830  873  916  959 1003 1046 1089 1132 1175 1219 1262 1305 1329 1353 1377 1401 1425 1464 1503 1541
[43] 1580 1619 1658 1658 1697 1735 1774 1813 1846 1879 1912 1945 1978 2023 2068 2112 2157 2202 2246 2290 2334
[64] 2378 2422 2446 2470 2493 2517 2541 2569 2597 2625 2653 2681 2705 2729 2752 2776 2800
```

---

### Part 3: Registration

This section automates looping through through and registering all atlas plates to images from the registration channel. It also allows for easy modification of registrations.

**`registration2()`** Provides a user friendly interface to alter registrations. It retains the same arguments from the `registration()` function in wholebrain. This function may be useful for those who already have their own established analysis pipelines. Check out the function help page for more details.


The schematic above illustrates the user-friendly console interface that allows for modification of the correspondence plates on a given atlas plate. The `regi_loop()` function integrates this user-friendly registration interface with the pipeline.

**Step 6.**

**`regi_loop()`** User friendly function to alter atlas to image registration. When run with defaults, this function will automatically create a list in the global environment called `regis`. It will loop through the atlas plates according to the user preferences. The user also has the option to save the global environment after every manual registration.

```
# Example run of automated registration loop

# Create filter for registration
filter<-structure(list(alim = c(50, 50),
                           threshold.range = c(50000, 60000L),
                           eccentricity = 999L,
                           Max = 1000,
                           Min = 150,
                           brain.threshold = 270L,
                           resize = 0.25,
                           blur = 7L,
                           downsample = 2))

# Run the autoloop
regi_loop(setup, savepaths, image_paths, autoloop = TRUE, filter = filter)
```

> Note: The function `filter_loop()` allows the user to modify the filter for registration or segmentation and loop through the references plates to check filter adjustments in images across the brain.

There are four different loop options:

1. `autoloop = TRUE` runs wholebrain’s first pass at registering all atlas plates. Registrations are stored in the *Registrations\_auto* folder. This is useful to check the quality of registrations across all atlas plates using current filter settings.
2. `reference = TRUE` loops through reference atlas plates only and displays the console interface.
3. The `touchup` argument is designed to edit a subset of atlas plates that have been previously registered

- `touchup = TRUE` runs a user friendly interface allowing user to enter which plates they want to fix.
- `touchup =` Numeric vector of plate numbers (integers) the user wants to fix.

4. Setting all the arguments above to `FALSE` will loop through all atlas plates and display the console interface.

> Tip: Save the global environment after every 2-3 registrations. The save time lengthens significantly as more registrations are stored in the `regis` list.

Once the `regi_loop()` function has been run for the first time, the `regis` argument must be set to the `regis` list automatically generated in the global environment.

For example:

```
# Loop through and modify only reference atlas plates
regi_loop(setup, savepaths, image_paths, regis = regis, filter = filter, reference = TRUE)
```

Below is an example of a registration before and after manual improvement:


---

### Part 4: Segmentation, duplicate cleanup, & forward warping

This section loops through all the images of the segmentation channel and automates the forward looping process. At the end of this section, you will have a mapped dataset!

**Step 7.**

**`filter_loop()`** This function takes a registration or segmentation filter and loops through all images in a partial brain or just the reference images in either the registration or segmentation channel. The `wholebrain::segment()` function is used to display the segmentation output. There is a console menu option for the user to adjust filter parameters if they need.

```
# Check and modify filter settings in the segmentaion channel
filter <- filter_loop(setup, image_paths, channel = "seg")
```

**Step 8.**

**`seg_loop()`** This function loops through the z images in the segmentation folder path. For a whole brain, every N images is segmented, where N is equal to `setup$seg_step`. For a partial brain, all the images in `setup$regi_z` are segmented.

```
# Run segmentation loop and store the output
segs <- seg_loop(setup, image_paths, filter)
```

> Note: If the imaging dataset is large, steps 8, 9, & 10 will be time intensive processes. Processing time will be printed after each of these functions are finished.

**Step 9.**

\*\*`clean_duplicates()` (W) \*\* Duplicate cell count clean up of segmentation output from the `seg_loop()` function. This step is only necessary for large imaging datasets where the z step size is smaller than the width of a cell body.

```
# Clean duplicate cell counts 
segs <- clean_duplicates(setup, segs, z_thresh = 10, compare_depth = 200)

# z_thresh - Threshold (in um) in the z-plane for the maximum z distance 
# before a cell is counted as another cell.

# compare_depth -  Comparision depth in (um). Compare a single image with adjacent 
# images up to 200 um posterior.
```

**Step 10.**

\*\*`forward_warp()` (W) \*\* Loop through all segmented cells and perform forward warp loop back onto atlas space using registration and segmentation data. There are user options for plotting a schematic plot and saving forward warp images. The function will automatically interpolate AP coordinate values of z images in between atlas plates and assign them to the dataset.

```
# Perform forward warp
dataset <- forward_warp(setup, savepaths, segs, regis)
```

Below are representative forward warp and schematic images:

**Forward warp image**

```
run_path("schematics/forwardwarp.PNG")
```


**Schematic plot**

> Note: `forward_warp()` will automatically clean up any cell counts that aren’t assigned a region ID.

---

### Part 5: Dataset manipulation and plotting:

Following Step 10, there are no more steps in the pipeline. The remaining functions are designed for easy manipulation and plotting of the dataset.

**`get_rois()`** Allows the user to enter a character vector of Allen Mouse Brain Atlas abbreviations of regions of interest (ROIs). A subset of the dataframe from the wholebrain dataset of just the ROIs (and their subregions) are returned.

`get_rois()` is especially useful in plotting cell count tables of regions of interest:

```
# Get dataset of just rois
rois <- get_rois(dataset, rois = c("ACB", "CEA", "BLA", "HIP"))

# Plot region cell counts in a table
quartz()
wholebrain::dot.plot(rois)
```

```
## [1] ""
```

**`get_sunburst()`** Generate a sunburst plot using a forward warped dataset.

```
# Plot sunburst plot using the get_sunburst() function
SMART::get_sunburst(dataset)
```

> Note: By setting the `rois` argument, the sunburst will display only ROI data. If the `parent` argument is set to `FALSE`, the base layer in sunburst will be the first ROI.

**`get_tree()`** Create a dataframe of hierarchical region cell count data.

The code below generates a dataframe of the hierarchy tree for the hypothalamus:

```
# Hierarchical dataframe of just the hypothalamus
tree <- get_tree(dataset, rois = "HY")
View(tree)
```

> Note: this dataframe may be useful for generating other heirarchical plots such as treemaps.

\*\*`glassbrain2()` \*\* A modified version of wholebrain::glassbrain(). New options include:

1. Gives the user an option to turn off the “jitter” in cell placement in original glassbrain function (the jitter gives a more space filled look).
2. Does not show the glass brain display when `high.res` is set to “OFF” (otherwise set to TRUE or FALSE). Setting high.res to TRUE or FALSE will render a high or low resolution atlas boundary

```
# Generate rgl object and plot glassbrain without 3D atlas boundaries
glassbrain <- glassbrain2(dataset, high.res = "OFF", jitter = FALSE) 

# visualize glass brain 
glassbrain
```

```
<html><head>
<TITLE>RGL model</TITLE>
</head>
<body onload="rgl.start();"> 

<div align="center">
```

```
<p id="debug">
You must enable Javascript to view this page properly.</p>
</div>

<br>Drag mouse to rotate model. Use mouse wheel or middle button
to zoom it.
<hr>
<br>
Object written from rgl 0.99.16 by writeWebGL.

</body>
</html>
```

> Tip: Combine the `get_rois()` function output with `glassbrain2()` to get a glass brain of just ROIs.

[1] Daniel Furth - @wholebrainsuite (2015). wholebrain: Functions to segment and register cells from microscopic image files with R. R package version 0.1.1.
