# Supplementary figures and images for: SMART: An Open-Source Extension of WholeBrain for Intact Mouse Brain Registration and Segmentation
Source: eNeuro. 2022 May 3;9(3):ENEURO.0482-21.2022. doi: 10.1523/ENEURO.0482-21.2022 (PMC9070730; doi:10.1523/ENEURO.0482-21.2022)

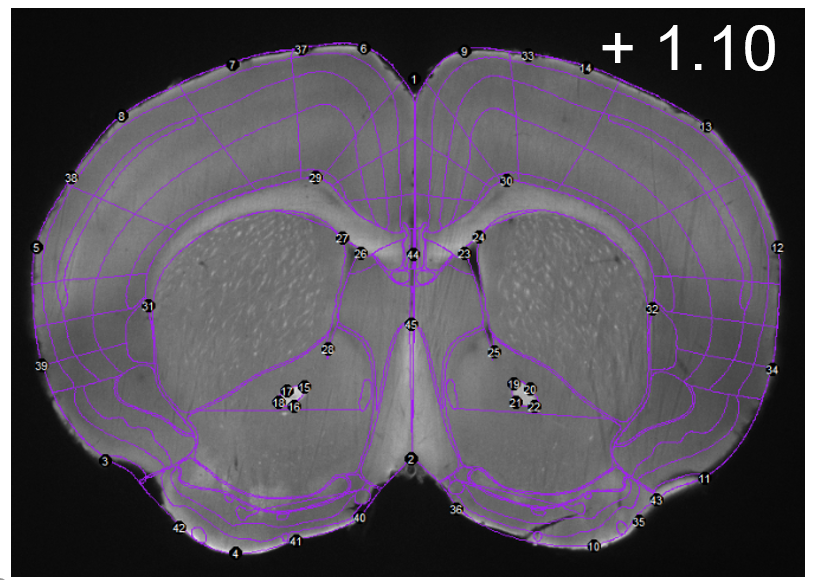

Supplement: Extended Data 1 — SMART code. Download Extended Data 1, ZIP file. [file enu-eN-OTM-0482-21-s03.zip › SMART/docs/schematics/after_registration.PNG]

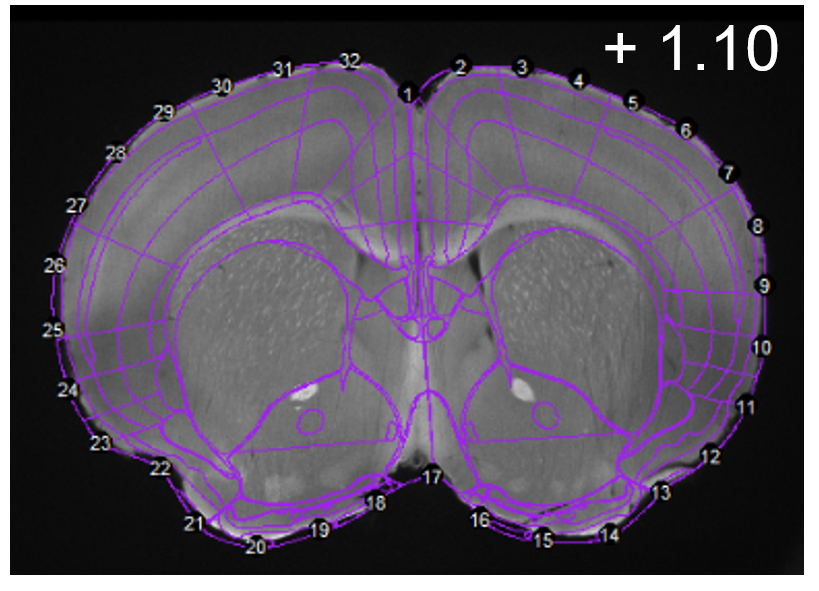

Supplement: Extended Data 1 — SMART code. Download Extended Data 1, ZIP file. [file enu-eN-OTM-0482-21-s03.zip › SMART/docs/schematics/before_registration.PNG]

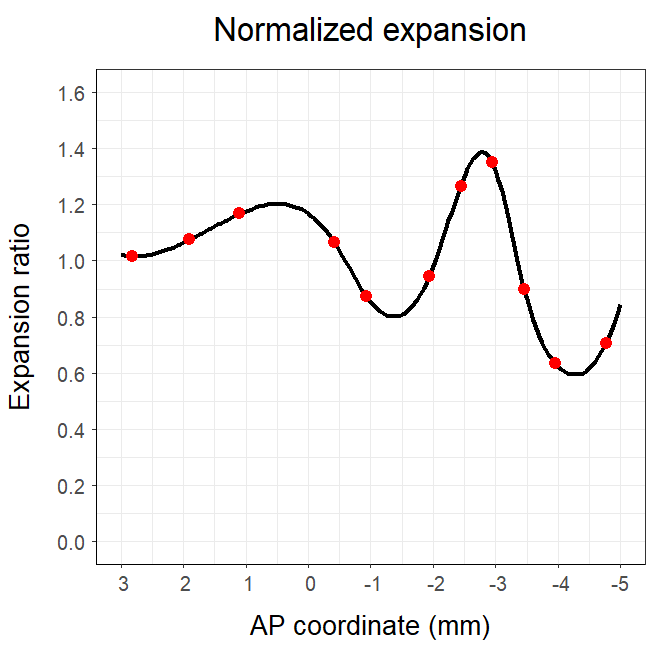

Supplement: Extended Data 1 — SMART code. Download Extended Data 1, ZIP file. [file enu-eN-OTM-0482-21-s03.zip › SMART/docs/schematics/brainmorph.PNG]

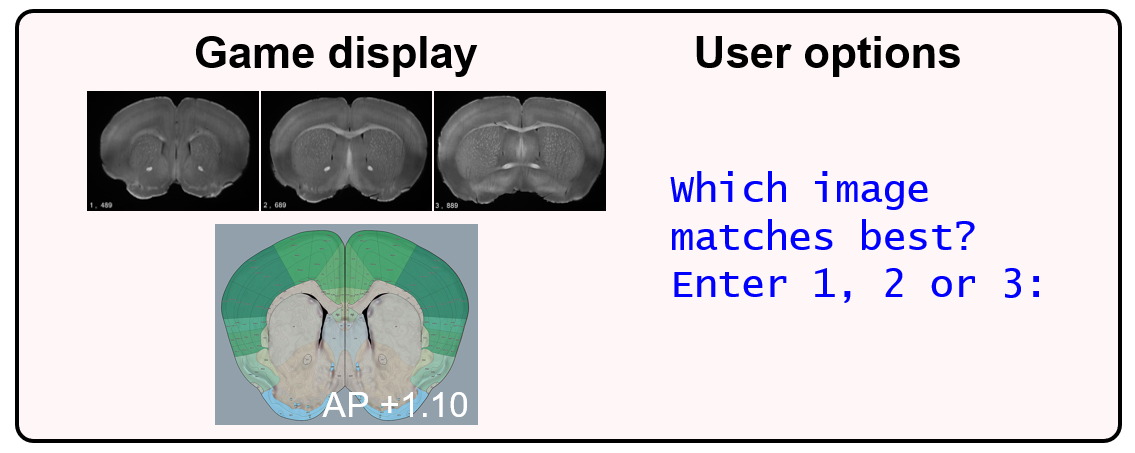

Supplement: Extended Data 1 — SMART code. Download Extended Data 1, ZIP file. [file enu-eN-OTM-0482-21-s03.zip › SMART/docs/schematics/choice_game_1.PNG]

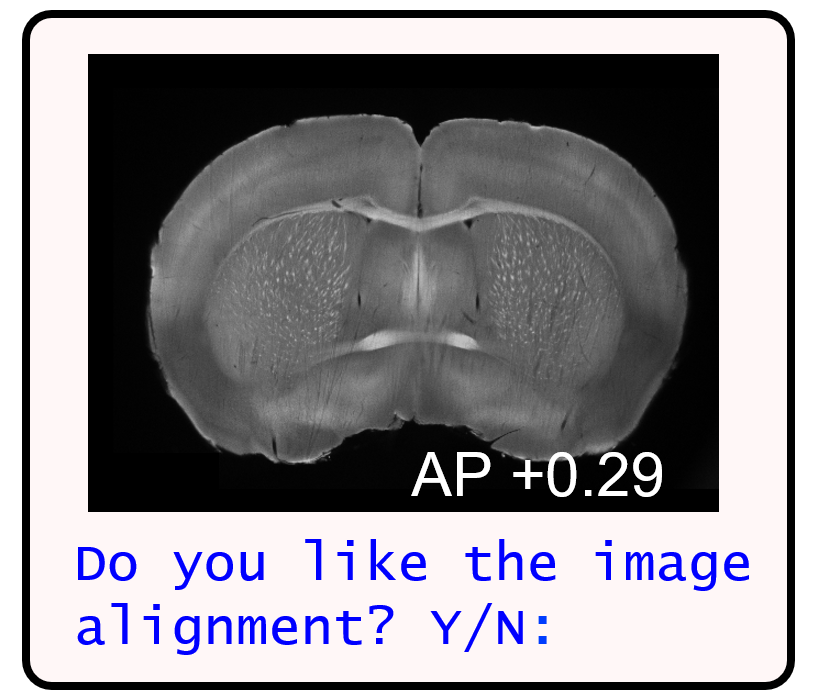

Supplement: Extended Data 1 — SMART code. Download Extended Data 1, ZIP file. [file enu-eN-OTM-0482-21-s03.zip › SMART/docs/schematics/choice_game_2.PNG]

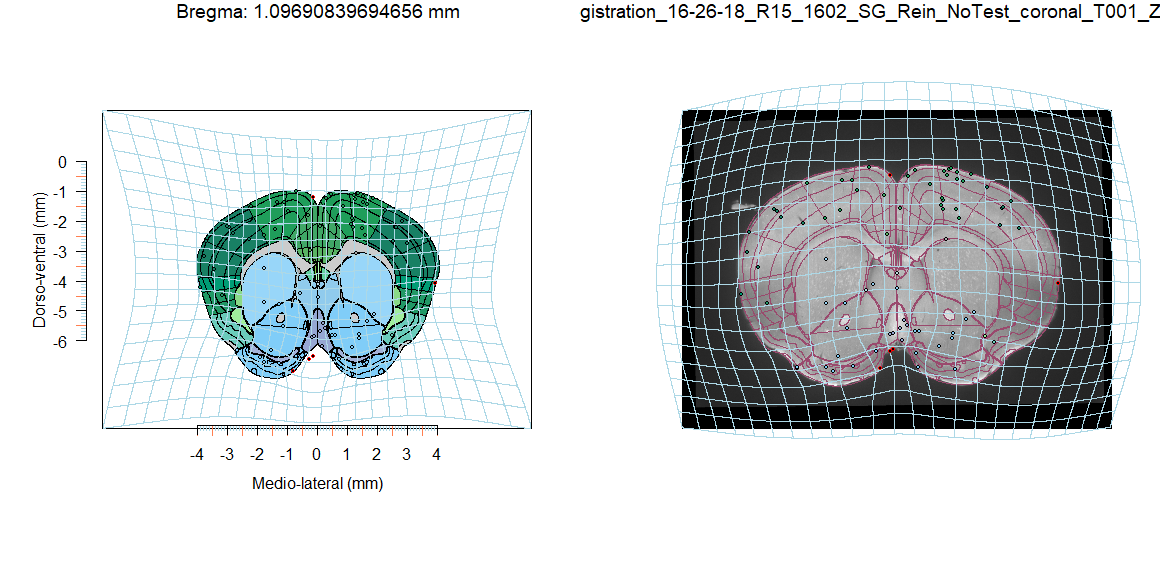

Supplement: Extended Data 1 — SMART code. Download Extended Data 1, ZIP file. [file enu-eN-OTM-0482-21-s03.zip › SMART/docs/schematics/forwardwarp.PNG]

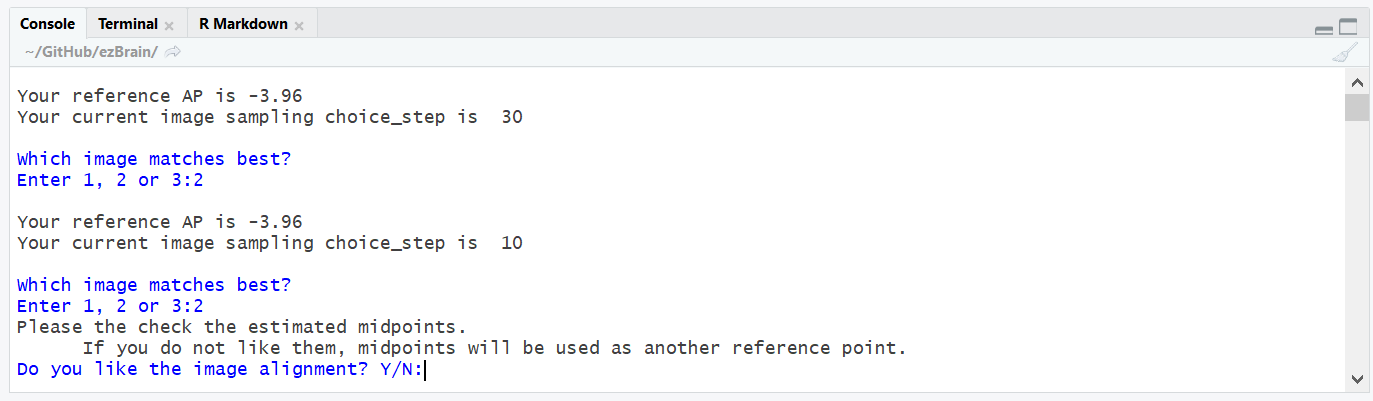

Supplement: Extended Data 1 — SMART code. Download Extended Data 1, ZIP file. [file enu-eN-OTM-0482-21-s03.zip › SMART/docs/schematics/midpoint_console.PNG]

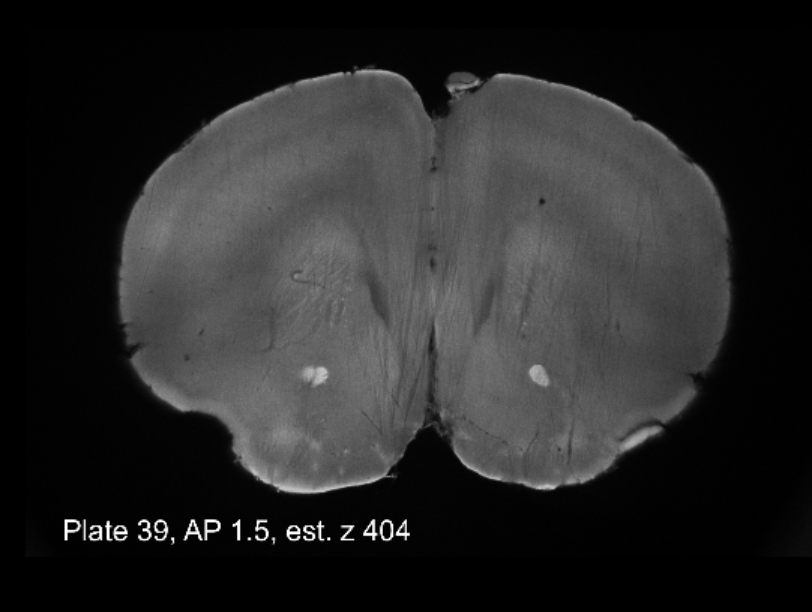

Supplement: Extended Data 1 — SMART code. Download Extended Data 1, ZIP file. [file enu-eN-OTM-0482-21-s03.zip › SMART/docs/schematics/midpoint_display.PNG]

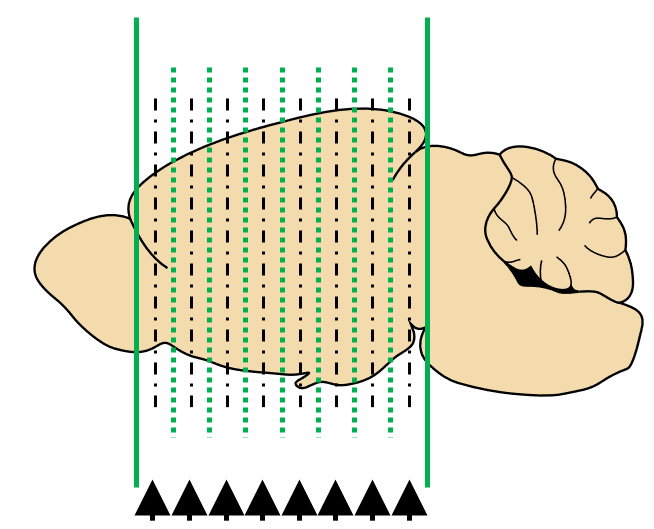

Supplement: Extended Data 1 — SMART code. Download Extended Data 1, ZIP file. [file enu-eN-OTM-0482-21-s03.zip › SMART/docs/schematics/midpoint_schem.PNG]

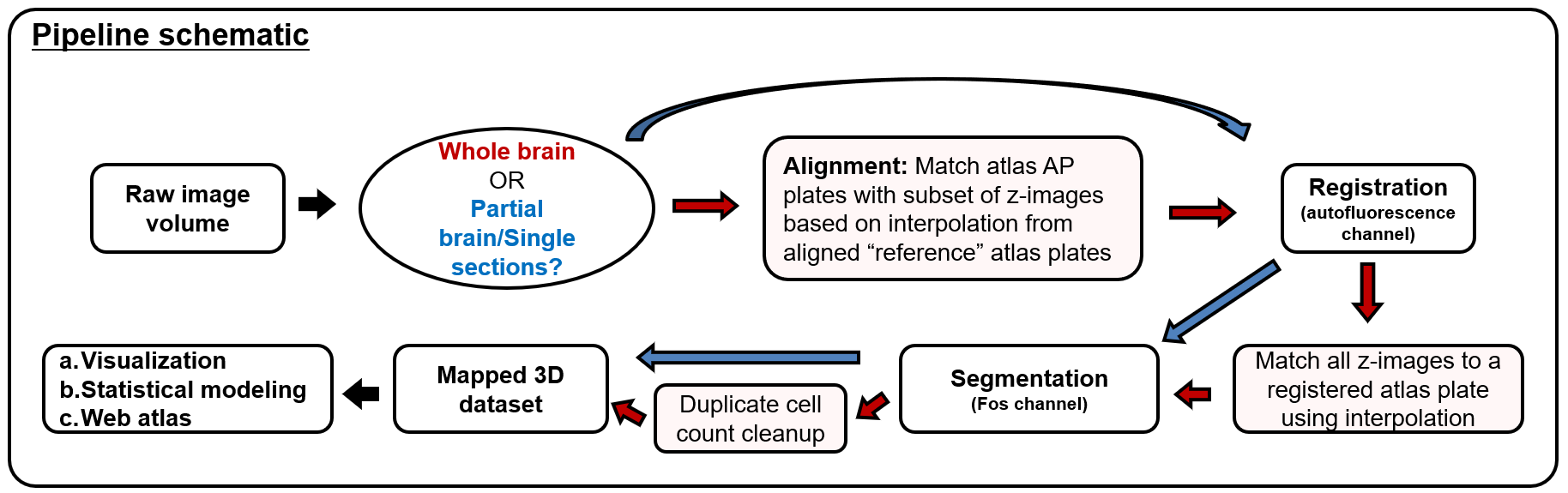

Supplement: Extended Data 1 — SMART code. Download Extended Data 1, ZIP file. [file enu-eN-OTM-0482-21-s03.zip › SMART/docs/schematics/pipeline_schematic.PNG]

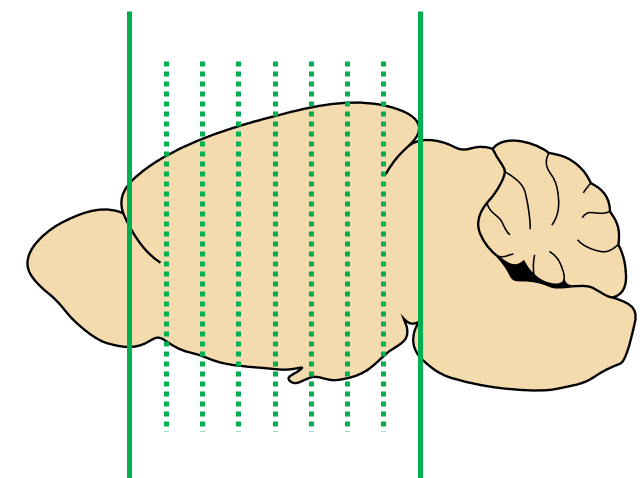

Supplement: Extended Data 1 — SMART code. Download Extended Data 1, ZIP file. [file enu-eN-OTM-0482-21-s03.zip › SMART/docs/schematics/Reference_atlas_schem.PNG]

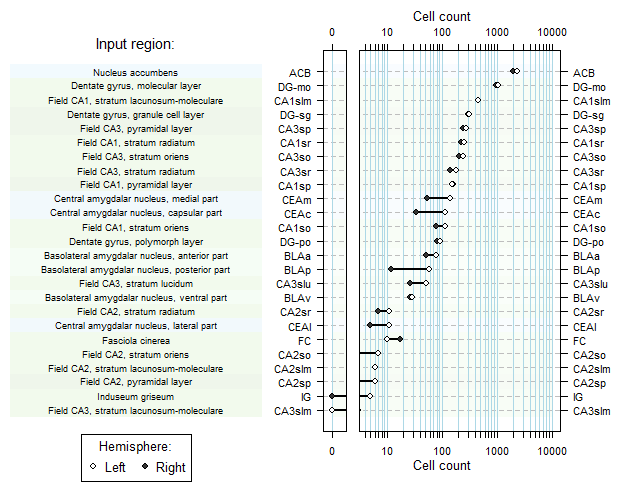

Supplement: Extended Data 1 — SMART code. Download Extended Data 1, ZIP file. [file enu-eN-OTM-0482-21-s03.zip › SMART/docs/schematics/region_plot.PNG]

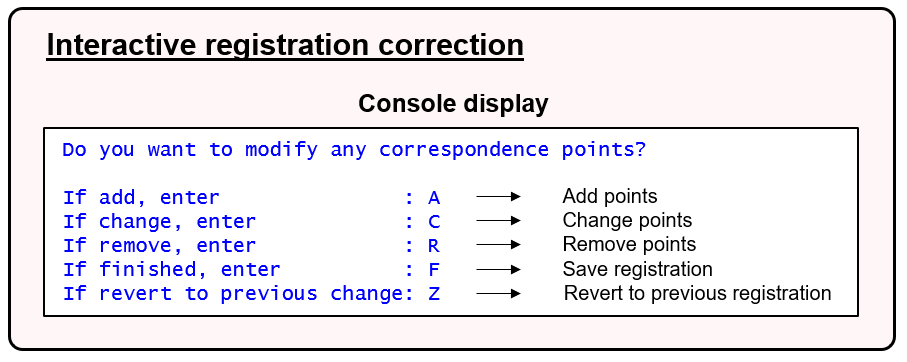

Supplement: Extended Data 1 — SMART code. Download Extended Data 1, ZIP file. [file enu-eN-OTM-0482-21-s03.zip › SMART/docs/schematics/registration_console.PNG]

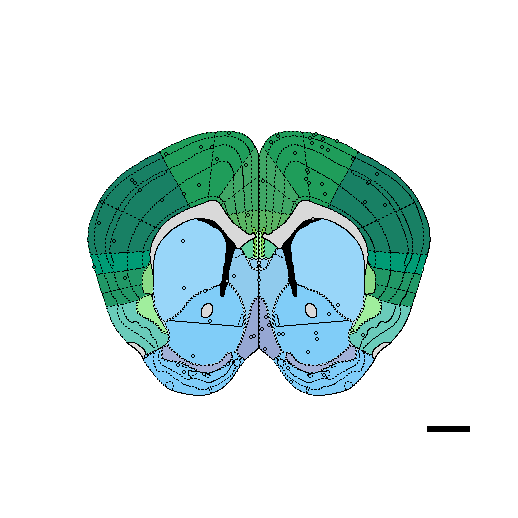

Supplement: Extended Data 1 — SMART code. Download Extended Data 1, ZIP file. [file enu-eN-OTM-0482-21-s03.zip › SMART/docs/schematics/schematic.PNG]
